# Brusatol Inhibits Esophageal Squamous Cell Carcinoma Tumorigenesis Through Bad-Mediated Mitochondrial Apoptosis Induction and Anti-Metastasis by Targeting Akt1

**DOI:** 10.3390/biom15060812

**Published:** 2025-06-04

**Authors:** Yao Ji, Xinxin Zhu, Yi Shi, Rui Fang, Yimeng Sun, Yurong Ruan, Liying Zhou, Yuanyuan Ge, Qichao Luo, Junyan Zhang, Junting Ma

**Affiliations:** 1 Department of Pharmacology, School of Basic Medical Sciences, Anhui Medical University, Hefei 230032, Chinaluoqichao@ahmu.edu.cn (Q.L.); 2 Department of Clinical Medicine, School of Medicine, Suizhou Vocational & Technical College, Suizhou 441300, China

**Keywords:** Brusatol, Akt1, mitochondria apoptosis, metastasis, esophageal squamous cell carcinoma

## Abstract

Esophageal squamous cell carcinoma (ESCC) is a prevalent malignancy characterized by poor prognosis and a deficiency of effective therapies. Brusatol (Bru), a bioactive component derived from *Brucea javanica*, exhibits potent anti-tumor activity. However, the pro-apoptotic and anti-metastatic effects of Bru in ESCC remain unclear. ESCC cells were incubated with Bru. The apoptotic status and metastatic capacities of the cells was measured by the Annexin V-FITC/PI, and wound-healing and transwell assays. Potential targets of Bru in ESCC were identified. The mechanisms by which Bru exerts its effects in ESCC cells were explored. Additionally, the typical 4-NQO-induced ESCC mouse model was employed to examine the anti-tumor effect of Bru in vivo. In this study, Bru was found to trigger mitochondria-mediated cell apoptosis (approximately 5.9- and 3.3-fold increases in the level of apoptosis at high concentrations (80 nM) in the KYSE30 and KYSE450 cells) and inhibit metastasis (49% wound closure decreases at high concentrations (80 nM) in both cells, compared to that in the DMSO group) in ESCC cells. In vivo, Bru significantly suppressed ESCC tumorigenesis. Notably, Bru interacts with Akt1, leading to a reduction in the phosphorylation level of Akt1 at Ser473. Consequently, this not only induced dephosphorylation of Bad at the Ser136 residue to promote mitochondrial apoptosis but also inhibited metastasis in ESCC cells. Bru promoted Bad-mediated mitochondrial apoptosis and inhibited the ESCC cell metastasis by targeting Akt1. Our results suggest Bru is a novel Akt1 inhibitor for inhibiting the progression of ESCC.

## 1. Introduction

Esophageal squamous cell carcinoma (ESCC), the predominant histologic subtype of esophageal carcinoma (EC), accounts for over 90% of EC cases and is associated with high global morbidity and mortality rates [1,2,3]. The absence of distinct clinical symptoms in early-stage esophageal cancer results in over half of patients being diagnosed at advanced stages. Patients with late-stage or metastatic esophageal cancer face poor prognosis and limited survival benefits [4]. Despite advancements in diagnostic technologies and therapeutic approaches, the prognosis for ESCC patients remains suboptimal, with a five-year survival rate of less than 20% [5,6]. The primary factors contributing to treatment failure include drug resistance and concurrent metastasis [7,8,9,10,11]. Even localized esophageal carcinomas frequently develop resistance to both chemotherapy and chemoradiotherapy [12]. Therefore, it is imperative to identify novel, safe, and effective therapeutic options for the prevention and management of ESCC.

Natural compounds have been broadly employed as therapeutic agents against various cancers due to their unique biochemical mechanisms and minimal side effects [13,14]. As a Traditional Chinese Medicine, *Brucea javanica* has been employed in the treatment of a range of inflammatory, oxidative stress-related diseases, and cancers [15,16]. In recent years, Brusatol (Bru), a compound isolated from *Brucea javanica*, has garnered significant attention as a potent anti-cancer agent [17]. Bru sensitizes a broad range of tumor cells, reduces chemoresistance, and enhances radiosensitivity in multiple cancers, including pancreatic carcinoma, lung cancer, and colorectal cancer [18,19,20]. Our previous study revealed that Bru induces ferroptosis in ESCC by inhibiting the nuclear factor erythroid 2-related factor 2 (NRF2) pathway [21]. However, we also observed an intriguing phenomenon in which Z-VAD-FMK (Z-VAD), a pan-caspase inhibitor, partially reversed the Bru-induced reduction in cell viability in ESCC cells, which suggests that Bru may induce apoptosis in ESCC cells [21]. Nevertheless, the mechanism underlying Brusatol-mediated apoptosis in ESCC remains unclear.

Dysregulation of apoptosis leads to cancer development [22]. Bad, a pro-apoptotic member of the Bcl-2 family, serves as a main regulator of apoptosis, mediating the cellular survival and apoptosis by regulating its phosphorylation status. When Bad is phosphorylated, its pro-apoptotic function is inhibited, which can contribute to cancer progression [23]. Conversely, dephosphorylated Bad can translocate to the mitochondrial outer membrane to form Bcl-2/Bad or Bcl-xL/Bad heterodimers, thereby triggering apoptosis [24]. A previous study revealed that Akt enhances Bad Ser136 phosphorylation to inhibit its pro-apoptotic function. Meanwhile, Bru has been shown to inhibit tumor proliferation and metastasis by targeting the PI3K/Akt pathway [25,26,27,28]. Hence, we hypothesize that the pro-apoptotic activity of Bru in ESCC may be linked to the deactivation of the Akt-phospho-Bad pathway.

In the current study, we comprehensively examined the pro-apoptotic and anti-metastatic activities of Bru, and precisely dissected its underlying mechanistic pathways in the context of ESCC. Our results indicated that Bru is a novel Akt1 inhibitor with significantly anti-ESCC potential by directly binding to Akt to inhibit the Akt1 pathway through the reduction of its phosphorylation. This mechanism not only promotes mitochondria-mediated apoptosis by suppressing the Akt1-phospho-Bad pathway but also inhibits the metastasis of ESCC cells. Our findings suggest that Bru may serve as a potential therapeutic agent for the treatment of ESCC and provide a reference for its further clinical application.

## 2. Materials and Methods

### 2.1. Reagents

Bru (TQ0211) from TopScience (Shanghai, China) was dissolved in dimethylsulfoxide (DMSO, GC203002) obtained from Servicebio (Wuhan, China). The Akt1 activator SC79 (SF2730) and Z-VAD (C1202) was sourced from Beyotime (Shanghai, China).

### 2.2. Cell Culture

KYSE30 and KYSE450 were obtained from Pricella and the China Center for Type Culture Collection (Wuhan, China), respectively, and were cultured in RPMI1640 medium (Bio-Channel, Nanjing, China) supplemented with 10% fetal bovine serum (FBS, Yeasen, Shanghai, China) and 1% penicillin/streptomycin (P/S) (C0222, Beyotime, Shanghai, China). Both cells were plated into 6 cm dishes, and incubated at 37 °C in a humidified atmosphere with 5% CO_2_. To determine the role of Bru in anti-ESCC activity, cells (60–70% confluence) were treated with Bru (20, 40, 80 nM) for 24 h or 48 h.

### 2.3. Apoptosis Assays

ESCC cells were treated with 20, 40, and 80 nM Bru, a combination of 40 nM Bru and 11 µM SC79, or 40 nM Bru plus 50 nM Bad shRNA for 24 h. ESCC cells were then assessed for apoptosis using an FACS Celesta flow cytometer (BD Celesta, Becton Dickinson, Franklin Lakes, NJ, USA) by utilizing the Annexin V-FITC Apoptosis Detection Kit (C1062S; Beyotime, Shanghai, China) following the manufacturer’s protocols. The apoptosis rate was quantified by using FlowJo 10.8.1 software.

### 2.4. Cell Counting Kit-8 (CCK-8) Assay

Adherent ESCC cells (5 × 10^3^ cells/well) in 96-well plates were exposed to 80 nM Bru, combined 80 nM Bru with 20 µM Z-VAD, or 11 µM SC79 for 24 or 48 h. Each well was then treated with 10 μL CCK-8 (C0038; Beyotime, Shanghai, China) at 37 °C for 2 h. OD values were examined on a multifunctional measuring instrument (Flash, SuperMax 3100, Shanghai, China) at 450 nm.

### 2.5. Wound-Healing Assay

KYSE30 and KYSE450 cells were cultured in 6-well plates to assess cell motility. The cells scratched with a germ-free 10 µL pipette tip were cultured for 24 h, with 20, 40, and 80 nM Bru treatment or combined 80 nM Bru with 11 µM SC79, to evaluate the extent of the wound healing. The wound closure areas were visualized under inverted microscope (Axiovert 25, Carl Zeiss, Jena, Germany) at the same location at 0 or 24 h. The relative distance of the borders was measured with ImageJ software (National Institutes of Health, Bethesda, MD, USA).

### 2.6. Transwell Assay

KYSE30 and KYSE450 cells were pre-treated with 20, 40, and 80 nM Bru, or a combination of 40 nM Bru and 11 µM SC79 for 24 h, and were subsequently resuspended and spread on the upper chamber (24-well insert chambers; LABSELECT, Hefei, China) precoated with Matrigel (C0372; Beyotime, Shanghai, China), and 700ul 1640 medium containing 10% FBS was added to the lower chamber. The chamber was incubated at 37 °C, 5% CO_2_ for 24 h, then removed. The cells were fixed with 4% paraformaldehyde (P0099, Beyotime, Shanghai, China), stained with 0.1% crystal violet (C0121, Beyotime, Shanghai, China), and counted by light microscope (Axiovert 25, Carl Zeiss, Jena, Germany).

### 2.7. Quantitative Real-Time Polymerase Chain Reaction (qRT-PCR)

The mRNA levels of target genes were assessed by RT-qPCR. Beyozol reagent (R0011, Beyotime, Shanghai, China) was applied to extract total RNA from treated KYSE30 and KYSE450 cells. cDNA was synthesized using a SuperMix for qPCR (11141ES60; Yeasen Biotechnology, Shanghai Co., Ltd., Shanghai, China). Subsequently, qRT-PCR analyses were conducted by Hieff^®^ qPCR SYBR Green Master Mix (11201ES08; Yeasen Biotechnology, Shanghai Co., Ltd., Shanghai, China). The mRNA expression at the following cycling settings: one initial cycle at 95 °C for 5 min followed by 40 cycles of 10 s at 95 °C, and 30 s at 60 °C. Relative mRNA expression was quantified by the 2^−ΔΔCt^ method. The primer sequences are listed in Table S1.

### 2.8. Mitochondria Isolation and Mitochondrial Membrane Potential (MMP) Analyses

To detect cytosolic cytochrome C (Cyto C), mitochondria were isolated from treated KYSE30 and KYSE450 cells using the Mitochondria Isolation Kit (C3601; Beyotime, Shanghai, China). The evaluation of MMP was conducted through JC-1 staining using the enhanced MMP assay kit with JC-1 (C2003S; Beyotime, Shanghai, China). All the experiments were performed according to the manufacturer’s instructions. The intensity of fluorescence was measured with ImageJ software (National Institutes of Health, Bethesda, MD, USA).

### 2.9. Network–Pharmacology Analysis for Bru

#### 2.9.1. Potential Target Screening

The Pubchem database (https://pubchem.ncbi.nlm.nih.gov/, accessed on 28 December 2024) was employed to obtain the corresponding 2D and 3D structures of Bru (CAS: 14907-98-3). Subsequently, we used the Pubchem database, PharmMapper database (https://www.lilab-ecust.cn/pharmmapper/submitfle.html, accessed on 2 January 2025), and BATMAN-TCM (http://bionet.ncpsb.org.cn/batman-tcm/, accessed on 2 January 2025 ) to retrieve Bru candidate targets. Furthermore, the hub gene targets for ESCC were predicted through DisGeNET (http://www.disgenet.org/, accessed on 28 December 2024), the Comparative Toxicogenomics Database (https://ctdbase.org/, accessed on 28 December 2024), and GeneCards (https://www.genecards.org, accessed on 28 December 2024). Common targets between Bru and ESCC were incorporated in the Venny-2.1 web tool (https://bioinfogp.cnb.csic.es/tools/venny/, accessed on 21 January 2025.).

#### 2.9.2. Protein-Protein Interaction (PPI) Network and Pathway Enrichment

The intersection genes of Bru and ESCC were uploaded to STRING (https://cn.string-db.org, accessed on 7 January 2025) to obtain PPI with the biological species (*Homo sapiens*) and the confidence level (greater than 0.9). The TSV files of the results were analyzed and the visual network of PPI was constructed using Cytoscape 3.8.0. We also used the DAVID database (https://david.ncifcrf.gov/, accessed on 2 January 2025) to analyze Gene Ontology (GO) terms and Kyoto Encyclopedia of Genes and Genomes (KEGG) pathways for intersection genes.

#### 2.9.3. Western Blotting (WB)

Western blotting was performed to detect the protein expression. WB was conducted as previously described [29]. Protein signals were determined following the manufacturer’s protocols using the Tanon™ ECL kit (180-501; Tanon, Shanghai, China). Information on the antibodies used in this paper is listed in Table S2. All results are derived from at least three independent biological replicates, and representative results are shown. Protein levels were quantified by densitometry using ImageJ software (National Institutes of Health, Bethesda, MD, USA), Original figures can be found in Supplementary Materials.

#### 2.9.4. Immunofluorescence (IF) Assay

Treated KYSE30 and KYSE450 cells were fixed with 4% paraformaldehyde, permeabilized with 0.1% Triton X-100, and incubated with 200 nM Mito-Tracker Red CMXRos (C1035; Beyotime, Shanghai, China) and rabbit anti-Bad antibody (R23582; 1:200; Zenbio, Chengdu, China) at 37 °C for 2 h. Subsequently, cells were incubated with a secondary antibody conjugated to Alexa Fluor 488 (511201; 1:1000; Zenbio, Chengdu, China) for 1 h. Cells were then treated with 2-(4-Amidinophenyl)-6-indolecarbamidine dihydrochloride (DAPI; C1005, Beyotime, Shanghai, China) for 5 min. Finally, photomicrographs of the mounted cells were captured using a Leica DC300F fluorescent microscope (Axiovert 25, Carl Zeiss, Jena, Germany). Information of the antibodies used in this paper is listed in Table S2.

#### 2.9.5. Drug Affinity Responsive Target Stability (DARTS) Assay

KYSE30 or KYSE450 cells were lysed in M-PER^®^ mammalian protein extraction with protease inhibitors. Cell lysates were equally divided into separate tubes and incubated for 1 h at room temperature (RT) with either DMSO or 400 nM Bru. Subsequently, Pronase (0, 0.5, and 1 μg/μL) was added to the incubated samples to carry out proteolysis at 25 °C for 1 h. The samples were then analyzed by WB assay. Protein levels were quantified by densitometry using ImageJ software (National Institutes of Health, Bethesda, MD, USA).

#### 2.9.6. Cellular Thermal Shift Assay (CETSA)

ESCC cell lysates were equally divided into six tubes and incubated with 1 μL of Bru (400 nM) or DMSO for 1h at RT. These tubes were then heated for 3 min at the specified temperatures and then cooled in ice for an additional 3 min. The cell lysates were centrifuged at 13,400× *g* for 20 min, and the supernatants were used for WB analysis. Protein levels were quantified by densitometry using ImageJ software (National Institutes of Health, Bethesda, MD, USA).

#### 2.9.7. Molecular Docking

The 3D structure of Akt1 protein and the SDF structure of Bru were obtained from the PDB database and PubChem databases, respectively. After removing water and adding hydrogen atoms to the Akt1 protein by AutoDock Tools 1.5.7 software, Molecular docking was conducted using AutoDock Vina, and visualized with PyMOL (TM-2.5.5). It is generally accepted that a binding energy of less than −5 indicates strong binding activity.

### 2.10. Animal Experiments

Ethical consent and experimental procedures were approved by the Anhui Medical University Laboratory Animal Centre (ethical approval code: LLSC20210774). Female C57BL/6 mice (4–5 weeks old, *n* = 6 group, Gem Pharmatech, Nanjing, China) were randomly allocated to four groups. The normal group and solvent control group (PG group) received water and water containing propylene glycol (100 μg/mL) starting from the first week. The 4-NQO group was treated with 4-NQO (100 μg/mL) from the first to the fifteenth week, and then treated with water from the 15th week. The Bru group were given water containing 4-NQO (100 μg/mL) from the first to the fifteenth week, and then administered Bru (1 mg/kg) via intraperitoneal injection three times a week starting at the fifteenth week. Body weight changes were monitored weekly. At the twenty-seventh week, the mice were euthanized using pentobarbital, and the whole esophageal tissues of mice in each group were fixed, and embedded for Hematoxylin and eosin (H&E) staining to detect the ESCC induction, Immunohistochemistry (IHC) to examine the level of p-Akt1 and p-Bad, and terminal deoxynucleotidyl transferase-mediated nick end labeling (TUNEL) staining to determine the level of cell apoptosis. In addition, the heart, liver, spleen, lung, and kidney were also fixed, and embedded for H&E staining to test for pathological alterations.

### 2.11. Tissue Staining

Tissue sections (5 μm) from each group were subjected to H&E staining (to examine the size of tumor lesions in esophageal tissues), TUNEL assay (to assess apoptotic cells within tumor lesions), and IHC (to evaluate the expression of p-Akt1 and p-Bad in the tumor lesions). H&E, IHC, and TUNEL assay were carried out by Servicebio (Wuhan, China). All sections were scanned using a Panoramic MIDI scanning microscope (3D Histech, Pannoramic MIDI, Budapest, Hungary).

### 2.12. Statistical Analysis

All assays were performed in at least three independent experiments. Before conducting statistical analysis, normality and homogeneity of variance tests were conducted first. Results are expressed as means ± standard error of the mean (SEM). The unpaired Student’s *t*-test or one-way ANOVA was performed to assess differences two groups or multiple groups by using GraphPad Prism version 8.4.2. *p* < 0.05 was considered statistically significant, with “ns” indicating not significant.

## 3. Results

### 3.1. Bru Induced Caspase-9/3-Dependent Apoptosis in ESCC Cells

**Figure 1:**
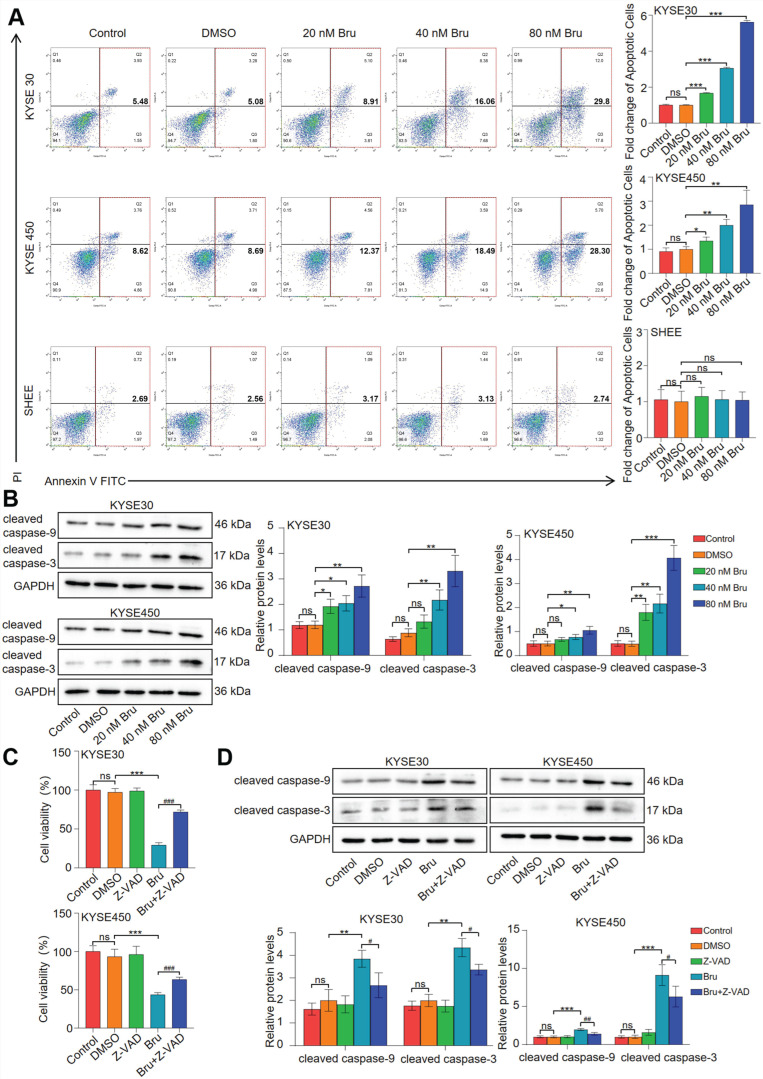
Bru activated caspase-dependent apoptosis in ESCC cells. KYSE30, KYSE450, and SHEE cells were exposed to various dosages of Bru (20, 40, 80 nM) for 24 h. (**A**) flow cytometry was employed to examine the apoptosis. (**B**) Protein levels of cleaved caspase-9 and c-caspase-3 were determined by WB assay. ESCC cells were treated by Z-VAD, Bru, and Z-VAD + Bru for 48 h. (**C**) CCK-8 assay was employed to measure cell viability. (**D**) Expression levels of c-caspase-9 and c-caspase-3 were examined by WB assay. For (**B**,**D**), the protein bands were quantified using ImageJ software. The data are presented as means ± SEM (*n* = 3). * *p* < 0.05, ** *p* < 0.01, *** *p* < 0.001 vs. DMSO group. ^#^ *p* < 0.05, ^##^ *p* < 0.01, ^###^ *p* < 0.001 vs. Bru group. ns, no significance.

To evaluate the function of Bru, KYSE30, KYSE450, and immortalized human esophageal epithelial cell line (SHEE) were incubated with various doses of Bru for 24 h. Compared with the DMSO group, the rate of cell apoptosis in the groups treated with 20, 40, and 80 nM Bru increased by approximately 1.8-, 3.2-, and 5.9-fold, respectively, in KYSE30 cells, while the cell apoptosis rate increased by approximately 1.4-, 2.1-. and 3.3-fold, respectively in KYSE450 cells. However, Bru did not induce apoptosis in SHEE cells (Figure 1A). Subsequently, WB assay indicated that the protein levels of cleaved caspase-9 (c-caspase-9) increased by 1.6-, 1.7-, and 2.3-fold, while cleaved caspase-3 (c-caspase-3) increased by 1.5-, 2.5-, and 3.7-fold in KYSE30 cells. In KYSE450 cells, the protein levels of c-caspase-9 increased by 1.3-, 1.5-, and 2.1-fold, while c-caspase-3 increased by 3.6-, 4.4-, and 8.2-fold (Figure 1B). However, the levels of cleaved caspase-8 protein did not exhibit significant changes (Figure S1A). Furthermore, Z-VAD-FMK pre-treatment enhanced the survival ability of Bru-treated KYSE30 and KYSE450 cells (Figure 1C). Additionally, the levels of c-caspase-9/c-caspase-3 protein were reduced in ESCC cells treated with Bru plus Z-VAD-FMK (Figure 1D). Collectively, these findings showed that Bru triggered apoptosis in ESCC cells through a caspase-9/3-dependent pathway.

### 3.2. Bru-Mediated Apoptosis Relied on Mitochondrial Dysfunction in ESCC Cells

**Figure 2:**
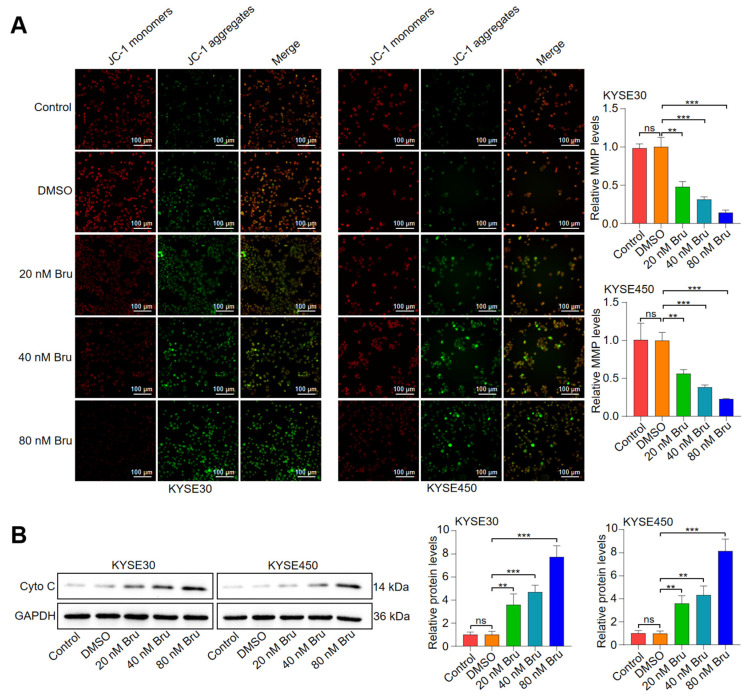
Bru-mediated apoptosis depended on mitochondrial dysfunction in ESCC cells. ESCC cells were incubated with DMSO, 20–80 nM Bru. (**A**) MMP changes were visualized via fluorescence microscopy, with quantification presented on the right. (**B**) The protein levels of Cyto C were monitored by WB assay and quantified using ImageJ software. The results are presented as means ± SEM (*n* = 3). ** *p* < 0.01, *** *p* < 0.001 vs. DMSO group. ns, no significance.

The mitochondrial specific caspase-9 was activated following Bru treatment, suggesting that Bru could induce mitochondrial dysfunction to trigger ESCC cells’ apoptosis. MMP loss and mitochondrial Cyto C release are key indicators of mitochondrial dysfunction. Thus, mitochondrial membrane depolarization and the level of Cyto C in the cytosol were measured using the JC-1 fluorescent probe and WB analysis, respectively. Figure 2A indicated that Bru treatment dramatically reduced the MMP in a dosage-dependent manner, with reductions of approximately 52%, 68%, and 86% in KYSE30 cells and 44%, 62%, and 77% in KYSE450 cells as the concentration increased. Furthermore, Bru administration resulted in a marked increase in cytosolic Cyto C level in a concentration-dependent manner, with increases of approximately 3.6-, 4.6-, and 7.7-fold in KYSE30 cells and 3.6-, 4.4-, and 8.2-fold in KYSE450 cells (Figure 2B). Collectively, the above findings showed that Bru triggered cellular apoptosis through affecting mitochondrial events in ESCC cells.

### 3.3. The Dephosphorylation of Bad Led to Bru-Mediated Apoptosis in ESCC Cells

**Figure 3:**
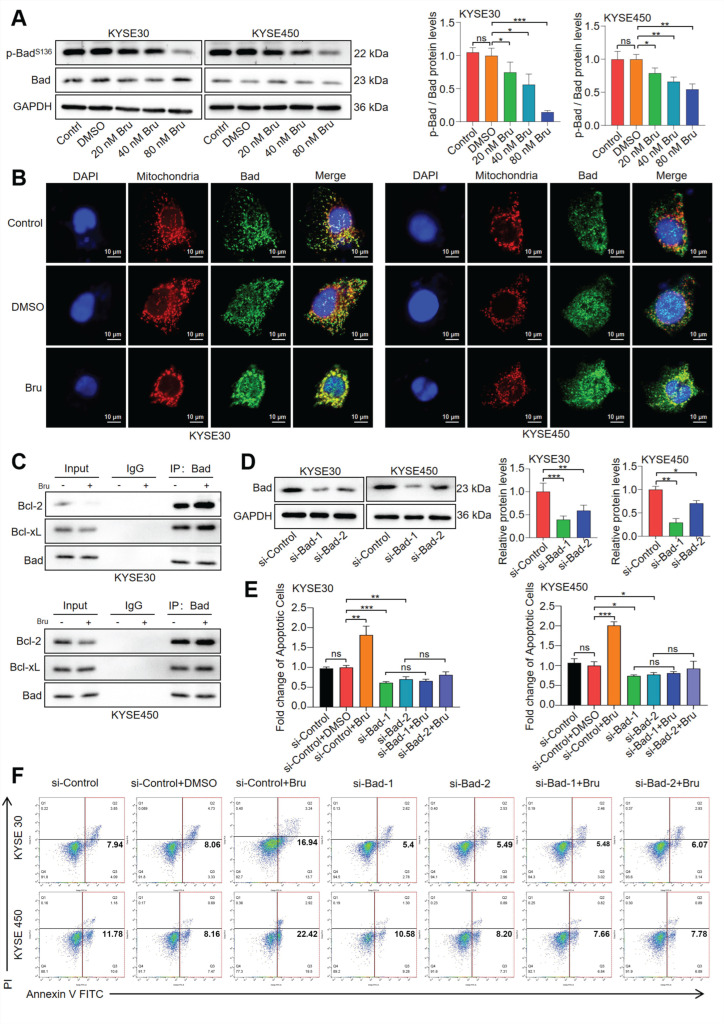
Bru-triggered apoptosis depended on the dephosphorylation of Bad in ESCC cells. (**A**) KYSE30 and KYSE450 cells were incubated with Bru (20–80 nM) for 24 h; the levels of pBad-S136 and Bad were analyzed by WB assay and quantified using ImageJ software. The results are presented as means ± SEM (n = 3). * *p* < 0.05, ** *p* < 0.01, *** *p* < 0.001 vs. DMSO group. ns, no significance (*p* > 0.05). (**B**) Bru-treated ESCC cells were examined for the mitochondrial localization of Bad by confocal microscopy. Green, red, and blue fluorescence indicate Bad, mitochondria, and nucleus, respectively. (**C**) WB assay revealed that Bru enhanced Bad binding to Bcl-2 or Bcl-xL. (**D**) ESCC cells were transfected with Bad siRNA; the levels of Bad were determined by WB assay and quantified using ImageJ software. The results are presented as means ± SEM (n = 3). * *p* < 0.05, ** *p* < 0.01, *** *p* < 0.001 vs. si-Control group. (**E**,**F**) Flow cytometry was employed to examine the apoptosis in treated ESCC cells and quantified using FlowJo software. The results are presented as means ± SEM (n = 3). * *p* < 0.05, ** *p* < 0.01, *** *p* < 0.001 vs. si-Control + DMSO group. ns, no significance (*p* > 0.05).

Abnormal dephosphorylation of Bad leads to its translocation to the outer mitochondrial membrane, where it dimerizes with Bcl-2 or Bcl-xL, thereby neutralizing its anti-apoptotic effects. This interaction subsequently enhances the release of Cyto C from the mitochondria and activates caspase-9 and caspase-3. As depicted in Figure 3A, Bru suppressed the phosphorylation level of Bad at Ser136 (S136) in a dosage-dependent manner by 25%, 44%, and 86% in KYSE30 cells, as well as by 21%, 34%, and 46% in KYSE450 cells. Additionally, IF assay indicated that Bru promoted the mitochondrial translocation of Bad (Figure 3B). Furthermore, Bcl-2 and Bcl-xL proteins were subjected to immunoprecipitation using the Bad antibody, and the protein content was detected via WB assay. We observed that the levels of both Bcl-2 and Bcl-xL proteins bound to Bad were upregulated following Bru treatment (Figure 3C). Moreover, we knocked down Bad using siRNA to research the function of Bru in the apoptosis of si-Bad ESCC cells (Figure 3D). The results indicated no significant difference in the apoptosis of si-Bad ESCC cells with or without Bru treatment. However, Bru treatment led to a 2.1-fold and 2.7-fold increase in apoptosis in KYSE30 and KYSE450 cells, respectively (Figure 3E,F). Together, these data showed that Bru induced mitochondria-mediated apoptosis by promoting the dephosphorylation of Bad in ESCC cells.

### 3.4. Bru Exhibited the Anti-Metastasis Activity in ESCC Cells

**Figure 4:**
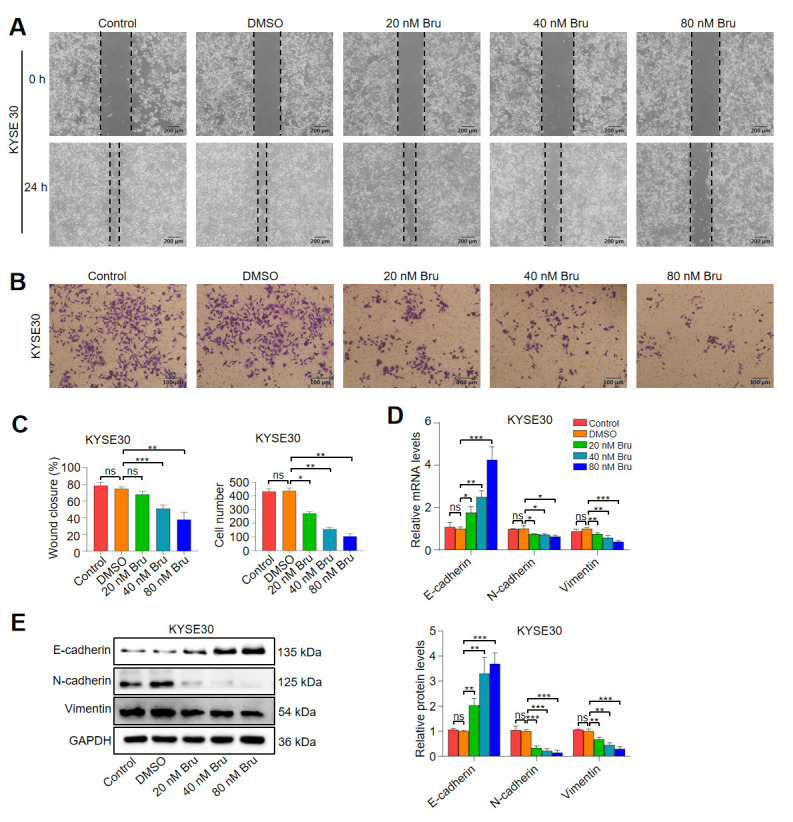
Bru suppressed migration and invasion of KYSE30 cells. ESCC cells were incubated with Bru (20–80 nM) for 24 h. (**A**–**C**) Cell migration and invasion were evaluated by wound-healing and transwell assays; quantification analysis was presented and quantified using ImageJ software. (**D**,**E**) mRNA and protein levels of E-cadherin, N-cadherin, and Vimentin were measured by qRT-PCR and WB assay with quantification using ImageJ software. The results are presented as means ± SEM (n = 3). * *p* < 0.05, ** *p* < 0.01, *** *p* < 0.001 vs. DMSO group. ns, no significance.

During the progression of tumor metastasis, migration and invasion are critical initial events [30]. KYSE30 and KYSE450 cells were exposed to varying doses of Bru (20 to 80 nM) for 24 h. Figure 4A–C and Figure S2A,B demonstrate that Bru significantly inhibited the migration (approximately 9%, 32%, and 49% wound closure decreases in KYSE30 cells, and approximately 12%, 23%, and 49% wound closure decreases in KYSE450 cells) and invasion (approximately 38%, 64%, and 76% reduction in the number of KYSE30 cells and approximately 29%, 66%, and 76% reduction in the number of KYSE450 cells) of KYSE30 and KYSE450 cells in a dose-dependent manner, suggesting that Bru could prevent ESCC cells from migrating and invading. Epithelial–mesenchymal transition (EMT) plays a crucial role in cell migration and invasion [31]. The loss of intercellular adhesion molecules, such as E-cadherin, alongside an increase in mesenchymal markers, including N-cadherin and Vimentin, are characteristic features of EMT [32]. As shown in Figure 4D, Bru-treated ESCC cells exhibited a decrease in N-cadherin mRNA levels by 25%, 28%, and 37%, and Vimentin mRNA levels by 24%, 42%, and 61%, while E-cadherin mRNA levels increased by 1.8-, 2.5-, and 4.3-fold. Consistent with the qRT-PCR results, WB analysis indicated that Bru treatment reduced N-cadherin protein levels by 67%, 78%, and 85%, and Vimentin protein levels by 32%, 55%, and 70%, while enhancing E-cadherin protein levels by 2.0-, 3.3-, and 3.7-fold in the KYSE30 cell line (Figure 4E). A similar trend was observed in the KYSE450 cell line (Figure S2C,D). These results indicated that Bru had a significant impact on inhibiting the metastasis of ESCC cells in vitro.

### 3.5. Akt1 Was a Direct Target of Bru

**Figure 5:**
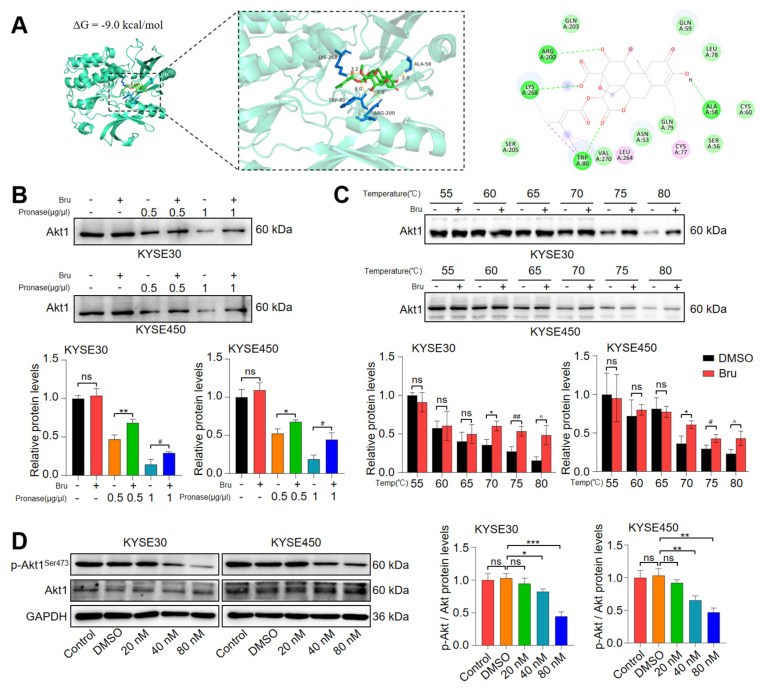
Akt1 was a direct target of Bru. (**A**) Molecular docking model between Bru and Akt1 based on the minimum binding energy. (**B**) KYSE30 and KYSE450 cells were incubated with or without Bru at varying doses of pronase for 1 h, and the protein level of Akt1 was assessed by WB assay. (**C**) KYSE30 and KYSE450 cells were incubated with or without Bru at RT for 1 h, then heated for 3 min at the specified temperatures and then cooled in ice for an additional 3 min. The protein level of Akt1 was assessed by WB assay. (**D**) The protein level of Akt1 and p-Akt1^Ser473^ was examined by WB assay. For H, the protein bands were quantified using ImageJ software. The results are presented as means ± SEM (n = 3). ^*^ *p* < 0.05, ** *p* < 0.01, *** *p* < 0.001, ^#^ *p* < 0.05, ^##^ *p* < 0.01, ^^^ *p* < 0.05 vs. DMSO group. ns, no significance.

To investigate the molecular mechanism of Bru in anti-ESCC activity, we retrieved 217 Bru-related genes from the Pubchem database, PharmMapper database, and BATMAN-TCM, and 6690 ESCC-related hub genes from DisGeNET, the Comparative Toxicogenomics Database, and GeneCards. Subsequently, common targets of Bru and ESCC were analyzed, revealing 160 gene targets that might be involved in the mechanism of Bru on ESCC (Figure S3A). Furthermore, GO and KEGG pathway enrichment analyses of 160 common targets between Bru and ESCC were performed using the DAVID 6.8 database. The results of GO analysis were primarily associated with biological processes (e.g., negative regulation of apoptotic process, the insulin-like growth factor receptor signaling pathway, peptidyl-tyrosine phosphorylation, protein autophosphorylation, the insulin receptor signaling pathway, protein phosphorylation, chromatin remodeling, the platelet-derived growth factor receptor-beta signaling pathway, the epidermal growth factor receptor signaling pathway, apoptotic process), cellular components (e.g., cytosol, cytoplasm, receptor complex, nucleus, nucleoplasm, membrane raft, mitochondrion, extracellular exosome, extracellular region, protein-containing complex), and molecular functions (e.g., nuclear receptor activity, histone H2AXY142 kinase activity, histone H3Y41 kinase activity, protein tyrosine kinase activity, zinc ion binding, enzyme binding, identical protein binding, ATP binding, sequence-specific DNA binding, transmembrane receptor protein tyrosine kinase activity) (Figure S3B). KEGG results revealed that the 160 targets participated in more than 144 KEGG pathways, including Pathways in cancer, Prostate cancer, Lipid and atherosclerosis, EGFR tyrosine kinase inhibitor resistance, Proteoglycans in cancer, Endocrine resistance, PI3K-Akt signaling pathway, Apoptosis, Chemical carcinogenesis—reactive oxygen species, and Toxoplasmosis (Figure S3C). In addition, 160 related target genes were imported into the STRING online database for PPI analysis. Figure S3D shows the PPI network of the 160 genes analyzed using Cytoscape 3.8.0, which displays 159 nodes and 1954 edges. Based on node degree values, the 10 high-ranking hub genes were shown as follows: Akt1, TNF, CASP3, ESR1, BCL2, EGFR, NFKB1, SRC, MMP9, and PTEN. Notably, Akt1 and TNF exhibited the highest number of connections, being linked to 93 additional proteins. Several studies have reported that Akt1 specifically phosphorylates Bad at Ser-136, and is also closely related to tumor metastasis [33,34,35]. Therefore, we hypothesized that Bru may target Akt1 in ESCC cells. To test this hypothesis, we first conducted a molecular docking study using the Autodock program, and found that the binding affinity of Bru to Akt1 was −9.0 kcal/mol, with hydrogen bonds with ALA58, TRP80, ARG 200, and LYS 268 (Figure 5A). Additionally, we employed DARTS and CETSA assays, two established techniques for assessing the binding of small molecules to target proteins, to evaluate the activity of Bru binding to the Akt1 protein. As illustrated in Figure 5B, WB analysis of DARTS samples indicated an increase in Akt1 stabilization during the proteolysis process, when KYSE30 and KYSE450 cell lysates were treated with Bru at a concentration of 400 nM. Furthermore, the CETSA assay revealed that Bru treatment enhanced the thermal stability of Akt1 at temperatures of 70 °C, 75 °C, and 80 °C, compared to the DMSO group (Figure 5C). Additionally, the expression level of Akt1 did not exhibit significant changes in response to increasing concentrations of Bru (Figure 5D). However, the phosphorylation level of Akt1 at Ser473 was downregulated by 8%, 20%, and 57% in KYSE30 cells and by 10%, 37%, and 55% in KYSE450 cells in a concentration-dependent manner following Bru treatment (Figure 5D). Collectively, these results indicate that Bru directly binds to Akt1 and dephosphorylates Akt1 at the S473 residue to inhibit the Akt1 pathway.

### 3.6. Bru Exhibited Mitochondria-Mediated Apoptosis via Inhibiting Akt1-Phospho-Bad Pathway in ESCC Cells

**Figure 6:**
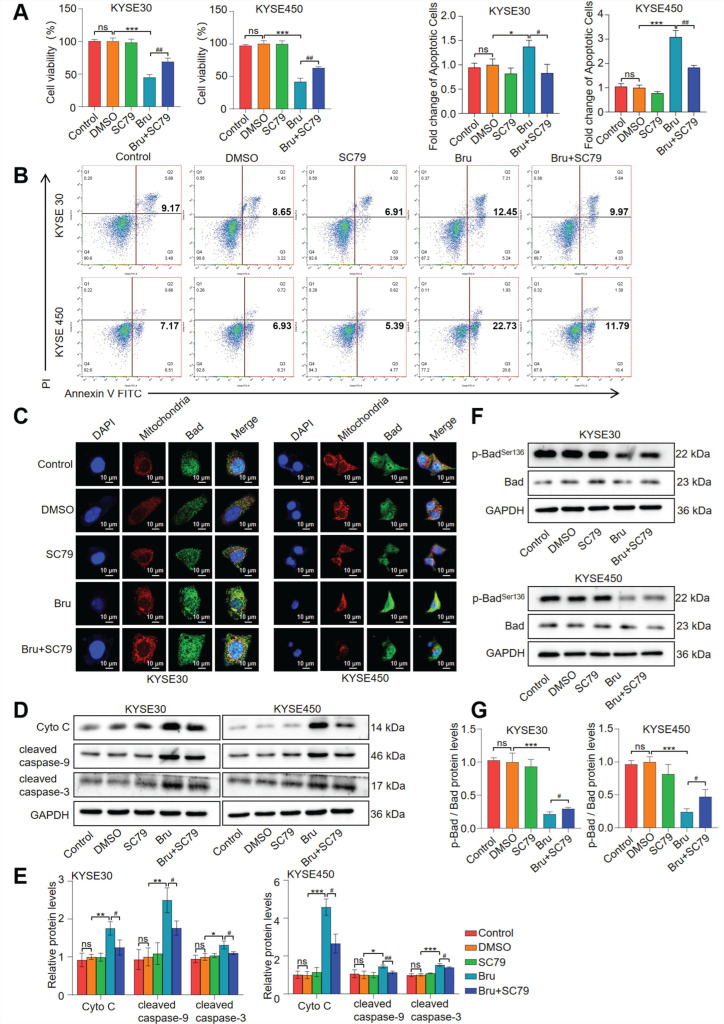
Bru induced mitochondria-mediated apoptosis via suppressing Akt1-phospho-Bad pathway in ESCC cells. ESCC cells were exposed to SC79, Bru, and SC79 + Bru. (**A**) CCK-8 analysis was used to detect cell viability. (**B**) the apoptosis was detected by flow cytometry. (**C**) Mitochondrial localization of Bad was visualized by confocal microscopy. Green, red, and blue fluorescence indicated Bad, mitochondria, and nucleus, respectively. (**D**–**G**) The protein levels of cytosolic Cyto C, c-caspase-9, c-caspase-3, pBad^Ser136^, and Bad were analyzed by WB assay and quantified using ImageJ software. The results are presented as means ± SEM (n = 3). * *p* < 0.05, ** *p* < 0.01, *** *p* < 0.001 vs. DMSO group. ^#^ *p* < 0.05, ^##^ *p* < 0.01 vs. Bru group. ns, no significance.

To research the function of the Akt1-phospho-Bad pathway in Bru-mediated apoptosis, we enhanced Akt1 activation using SC79, an Akt1 activator [36,37]. Our data indicated that SC79 alleviated the Bru-induced reduction in ESCC cell viability (*p* < 0.01, Figure 6A). Furthermore, SC79 reversed the increase in apoptosis triggered by Bru in both ESCC cell lines (*p* < 0.05, *p* < 0.01, Figure 6B). Concurrently, SC79 significantly reduced MMP and the mitochondrial translocation of Bad following Bru treatment (Figure 6C and Figure S4). Additionally, co-treatment with Bru and SC79 caused a downregulation of the expression levels of cytosolic Cyto C and c-caspase-9/c-caspase-3, compared to cells treated with Bru alone (*p* < 0.05, *p* < 0.01, Figure 6D,E). Moreover, SC79 pretreatment notably reversed the decrease in p-Bad^Ser136^ protein levels in Bru treated ESCC cell lines (*p* < 0.05, Figure 6F,G). These findings indicated that the Akt1-phospho-Bad pathway, activated by SC79, effectively inhibited Bru-induced apoptosis in ESCC cells.

### 3.7. Activation of Akt1 Pathway Attenuated the Anti-Metastasis Activity of Bru in ESCC Cells In Vitro

**Figure 7:**
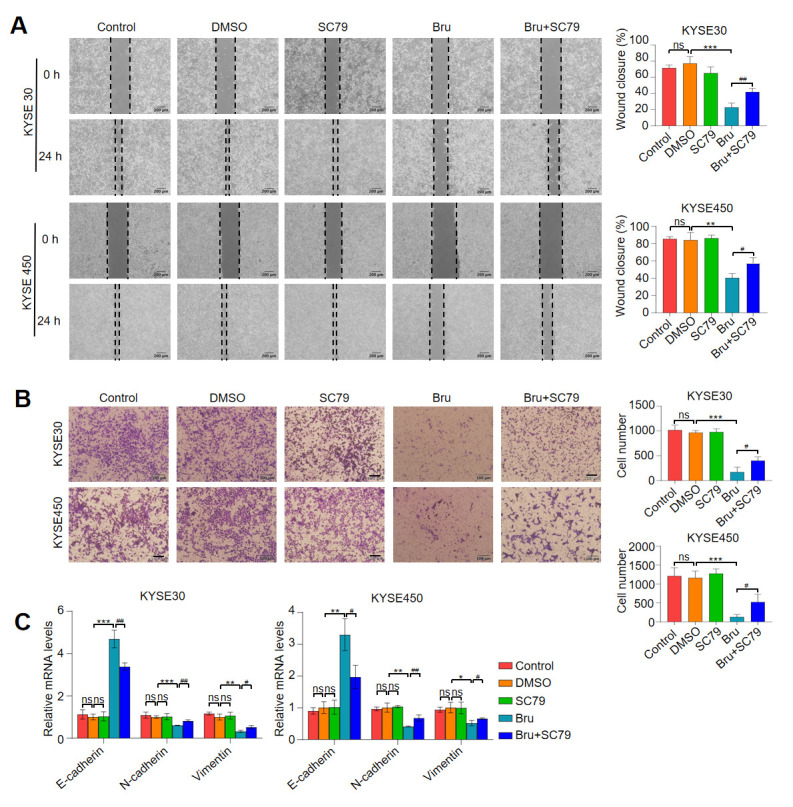
Activation of Akt1 pathway attenuated the anti-metastasis capacity of Bru in ESCC cells in vitro. KYSE30 and KYSE450 cells were incubated with SC79, Bru, and SC79 + Bru. (**A**,**B**) The cell migration and invasion were analyzed using wound-healing and transwell assays. Quantification analysis is presented on the right. (**C**) The mRNA levels of E-cadherin, N-cadherin, and Vimentin were measured by qRT-PCR assay. The results are shown as means ± SEM (n = 3). * *p* < 0.05, ** *p* < 0.01, *** *p* < 0.001 vs. DMSO group. ^#^ *p* < 0.05, ^##^ *p* < 0.01 vs. Bru group. ns, no significance.

To elucidate the role of the Akt1 pathway in the inhibition of metastasis in ESCC cells induced by Bru, we utilized the Akt1 activator SC79. The wound-healing assay demonstrated that pre-treatment of ESCC cells with SC79 significantly diminished the migration suppression induced by Bru in both KYSE30 and KYSE450 cell lines (*p* < 0.05, *p* < 0.01, Figure 7A). Furthermore, pre-treatment with SC79 markedly reduced the Bru-mediated inhibition of ESCC cell invasion (*p* < 0.05, Figure 7B). Additionally, SC79 pretreatment notably reversed Bru-induced downregulation of N-cadherin and Vimentin gene expressions and upregulation of E-cadherin gene expression in both ESCC cell lines (*p* < 0.05, *p* < 0.01, Figure 7C). Collectively, the activation of the Akt1 pathway by SC79 effectively counteracts Bru-induced inhibition of ESCC metastasis.

### 3.8. Bru Retarded ESCC Tumorigenesis

**Figure 8:**
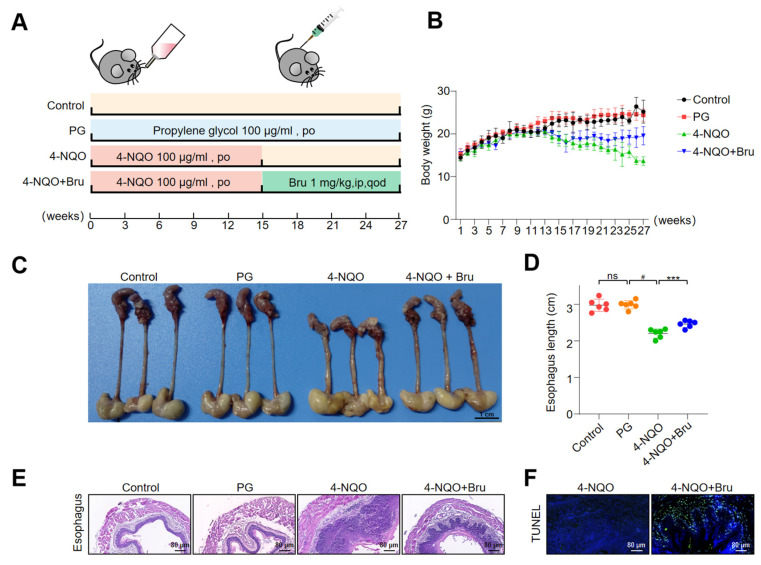

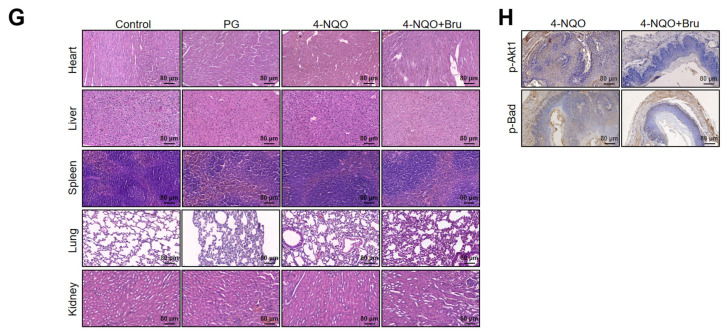
Bru inhibited ESCC tumorigenesis. (**A**) Schematic illustration of experimental design to measure the therapeutic effects of Bru in vivo. Drug administration dosages were indicated (n = 6). (**B**) Body weight of mice under different treatments. (**C**) Representative images of mouse esophagus. (**D**) The length of the mouse esophagus. (**E**) Histological analysis of the mouse esophagus with representative H&E images, 800× magnification. (**F**) Tumor cell apoptosis detected by DeadEnd^TM^ Colorimetric TUNEL System kit, with green fluorescence indicating TUNEL-positive (apoptotic) cells. (**G**) Histological analysis of the heart, liver, spleen, lung, and kidneys with representative H&E images, 800× magnification. (**H**) Protein levels of p-Akt1 and p-Bad in the esophageal tissues of mice in the 4-NQO group and the 4-NQO + Bru group were assessed by IHC. Representative images were shown, 800× magnification. *** *p* < 0.001 vs. 4-NQ group. ^#^ *p* < 0.05 vs. PG group. ns, no significance.

To study the anti-tumor function of Bru, we employed a 4-NQO-induced ESCC mouse model that mimics the progression of human ESCC. The female C57/BL6 mice were treated with 4-HQO for 15 weeks followed by 12 weeks of treatment with vehicle or Bru (Figure 8A). As shown in Figure 8B, Bru treatment displayed a marked reduction in the sign of weight loss, compared to the 4-HQO group. Meanwhile, the esophagus tissue in the 4-NQO group exhibited thickening, roughness, white patches, and palpable protuberances, which were significantly ameliorated in the 4-NQO + Bru group (Figure 8C). Notably, the length of the esophagus was obviously increased in the 4-NQO + Bru group (*p* < 0.001, Figure 8D). Furthermore, H&E staining demonstrated a remarkable reduction in the volume of tumor lesions in the esophageal tissues of mice treated with Bru (Figure 8E), and the TUNEL assay revealed a significantly higher number of apoptotic cells in the 4-NQO + Bru group than in the 4-NQO group (Figure 8F). These data indicate that Bru could reduce the formation of esophageal lesions induced by 4-NQO. Additionally, IHC analysis revealed a significant reduction in the phosphorylation levels of Akt1 and Bad in the tumor lesions of the 4-NQO + Bru group compared to the 4-NQO group (Figure 8H). Importantly, no pathological alterations were observed in the heart, liver, spleen, lung, or kidney tissues of the mice in the Bru treatment groups (Figure 8G), indicating no apparent toxicity from Bru treatment. Collectively, these findings confirm that Bru mitigates the progression of ESCC in vivo.

## 4. Discussion

ESCC, a malignant tumor characterized by unfavorable prognosis and high mortality, has garnered increasing attention [38]. ESCC originates from the squamous epithelium of the esophagus. The initial step in carcinogenesis involves the accumulation of genetic mutations or alterations that disrupt normal cellular functions and drive aberrant cell proliferation [39]. This process may lead to the formation of precancerous lesions, such as squamous dysplasia or carcinoma in situ, which can progress to invasive carcinoma if left untreated [40]. Currently, there is enormous progress in therapeutic methods, but effective treatment options for ESCC are not sufficient, and improvements in prognosis are still limited [41]. Hence, to explore efficacious therapeutic strategies for ESCC treatment is urgent.

Natural extracts are crucial sources for the discovery, optimization, and development of new drugs, and many natural plant extracts, such as paclitaxel, camptothecin, vinblastine, and podophyllotoxin, have been developed as clinical anti-tumor drugs. Bru is a quassinoid which has been developed as an experimental treatment for multiple cancers, including lung cancer, bladder cancer, and thyroid cancer [42,43,44]. In other studies, Bru has demonstrated pro-apoptotic and anti-metastatic activity in pancreatic cancer [45]. This study explores whether Bru has similar function on ESCC, specifically promoting ESCC cells’ apoptosis and inhibiting their metastasis. Our results show that Bru triggers mitochondria-mediated apoptosis and inhibits ESCC cell’s metastasis. Additionally, Bru significantly impedes ESCC progression in vivo without causing toxicity to other organs such as liver, lungs, or kidneys. These findings suggest that Bru could be an effective drug candidate for ESCC treatment, offering good efficacy with limited side effects. Therefore, the molecular mechanisms by which Bru suppresses ESCC through its pro-apoptotic and anti-metastatic activities should be further explored to enhance the confidence level of its potential clinical applications.

Dysregulation of apoptosis is a common phenomenon in cancer cells [22]. As a pro-apoptotic protein, Bad plays a fundamental role in the tumorigenesis of many human cancers [46]. Previous reports showed that Bad expression is downregulated in ESCC tissues, and dephosphorylated Bad, induced by Pim-3 depletion, can induce apoptosis to inhibit ESCC progression [47]. Our results demonstrated that Bru decreased the phosphorylation of Bad at Ser136, facilitating its mitochondrial translocation and the formation of Bcl-2/Bad or Bcl-xL/Bad heterodimers. This, in turn, promoted the release of mitochondrial Cyto C and the activation of caspase-9/3, ultimately triggering the apoptosis of ESCC cells. Several kinase proteins or protein complexes, such as ribosomal protein S6 kinase, cAMP dependent protein kinase, AKT, and IκB kinase complex, are capable of phosphorylating Bad at distinct regulatory residues (Ser26, Ser112, Ser136, and Ser155) to maintain its cytoplasmic sequestration and thus attenuate cell apoptosis [48,49,50,51]. Our findings indicated that Bru inhibited Akt1 phosphorylation, keeping Bad in a non-phosphorylated from. Consequently, Bru induced mitochondria-medicated apoptosis in ESCC cells.

Bru has been reported as a vital NRF2 inhibitor which specifically diminishes its expression through promoting its ubiquitin-mediated degradation [17]. Studies have shown that Bru is commonly used to research the role of some proteins or noncoding RNA in various disease by modulation of the NRF2 pathway, but it also exhibits promising anticancer potential through the NRF2-mediated defense mechanism [44,52]. However, there are very few direct binding proteins of Bru, which results in unclear anti-tumor mechanisms and restricts its further development. A previous study revealed that Bru directly interacts with S-phase Kinase-Associated Protein 1, impairing the proliferation and metastasis of non-small cell lung cancer [42]. In this study, we employed DARTS, CETSA, and molecular docking assays to identify the binding proteins of Bru, demonstrating that Bru directly binds to Akt1, leading to the dephosphorylation Akt1-Ser473 without affecting its expression. While several studies have indicated that Bru can inhibit the PI3K/Akt pathway [26], our results elucidate the specific and detailed mechanism of the PI3K-Akt1 pathway mediated by Bru.

After detaching from their original sites of formation, cancer cells migrate through the blood or lymphatic systems and subsequently form new tumors in other parts of the body, accounting for up to 90% of cancer fatalities [53,54,55]. In this study, Bru significantly impaired the migration and invasion of ESCC cells. EMT contributes to cancer cells metastasis. During this complex process, epithelial cells undergo phenotypic changes, acquiring mesenchymal characteristics that facilitate invasion and migration [56]. These cellular transformations are marked by the downregulation of epithelial markers such as E-cadherin and the upregulation of markers like N-cadherin and vimentin [57]. qRT-PCR and WB assay demonstrated that Bru impeded the EMT process by upregulating E-cadherin and decreasing N-cadherin and Vimentin. Furthermore, the activation of the Akt1 pathway by SC79 could attenuate the anti-metastasis function of Bru in ESCC cells. Therefore, Bru markedly inhibited ESCC metastasis via inhibiting the Akt1 pathway.

## 5. Conclusions

In summary, our study demonstrated that Bru, a novel Akt1 inhibitor, effectively targeted Akt1 protein and suppressed its phosphorylation at Ser473 residues, which not only promoted mitochondria-mediated apoptosis via inhibiting the Akt1-phospho-Bad pathway in vitro and in vivo, but also impeded the ESCC cell’s metastasis via suppressing the Akt1 pathway in vitro. Our findings strongly support its further development as a promising candidate for clinical trials in ESCC patients. However, there are still some limitations in this study. The in vivo anti-metastatic efficacy of Bru remains uninvestigated. Additional animal models and clinical trials are required to comprehensively assess the safety and antitumor activity of Bru as a monotherapy or in combination with chemotherapeutic agents.

## Data Availability

The datasets used and/or analyzed during the current study are available from the corresponding author on reasonable request.

## Supplementary Materials

The following supporting information can be downloaded at: https://www.mdpi.com/article/10.3390/biom15060812/s1, Figure S1: The effect of Bru on the expression of cleaved caspase-8; Figure S2: The effect of Bru on the migration and invasion of KYSE450 cells; Figure S3. Network pharmacology analysis of Bru; Figure S4. The effect of SC79 plus Bu on MMP of ESCC cells; Table S1: Sequences of the primers used in the real-time PCR; Table S2: Antibody list.

## Abbreviations

Akt1	Akt serine/threonine kinase 1 gene
Bcl-2	B-cell lymphoma-2
Bru	Brusatol
CCK8	Cell Counting Kit-8
CETSA	Cellular thermal shift assay
Cyto C	Cytochrome C
DARTS	Drug affinity responsive target stability
EC	Esophageal carcinoma
EMT	Epithelial–mesenchymal transition
ESCC	Esophageal squamous cell carcinoma
FBS	Fetal bovine serum
GO	Gene ontology
H&E	Hematoxylin and eosin
IF	Immunofluorescence
IHC	Immunohistochemistry
IP	Immunoprecipitation
KEGG	Kyoto Encyclopedia of Genes and Genomes
MMP	Mitochondrial membrane potential
NRF2	Nuclear factor erythroid 2-related factor 2
PPI	Protein-protein interaction
qRT-PCR	Quantitative real-time polymerase chain reaction
SDS	Sodium dodecyl sulfate
WB	Western blot
Z-VAD	Z-VAD-FMK
