# Supplementary material for: Brusatol Inhibits Esophageal Squamous Cell Carcinoma Tumorigenesis Through Bad-Mediated Mitochondrial Apoptosis Induction and Anti-Metastasis by Targeting Akt1
Source: Biomolecules. 2025 Jun 4;15(6):812. doi: 10.3390/biom15060812 (PMC12191141; doi:10.3390/biom15060812)
Supplement: Supplementary file 1 [file biomolecules-15-00812-s001.zip › 5.23 Supplementary Figures.pdf]

Supplementary materials

Brusatol Inhibits Esophageal Squamous Cell Carcinoma Tumorigenesis Through Bad-Mediated Mitochondrial Apoptosis Induction and Anti-Metastasis by Targeting Akt1

Yao Ji [1,2, †], Xinxin Zhu [1, †], Yi Shi[1], Rui Fang[1], Yimeng Sun[1], Yurong Ruan[1], Liying Zhou[1], Yuanyuan Ge[1], Qichao Luo[1], Junyan Zhang[1,*], and Junting Ma[1,*]

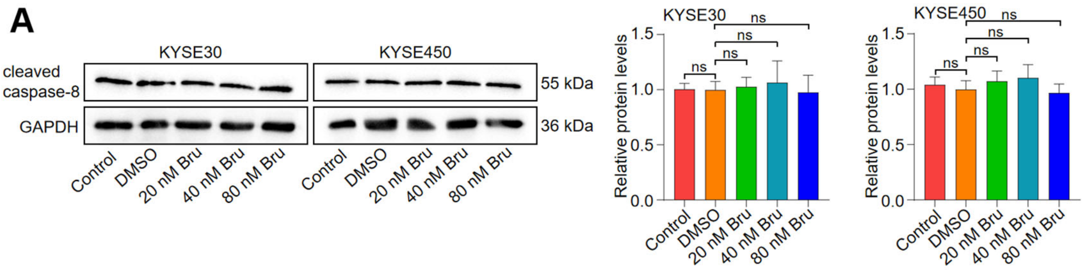

Figure S1 The effect of Bru on the expression of cleaved caspase-8. (A) Expression levels of cleaved-caspase-8 was examined by WB assay. The protein bands were quantified using ImageJ software. The data was presented as means ± SEM (n=3). ns, no significance.

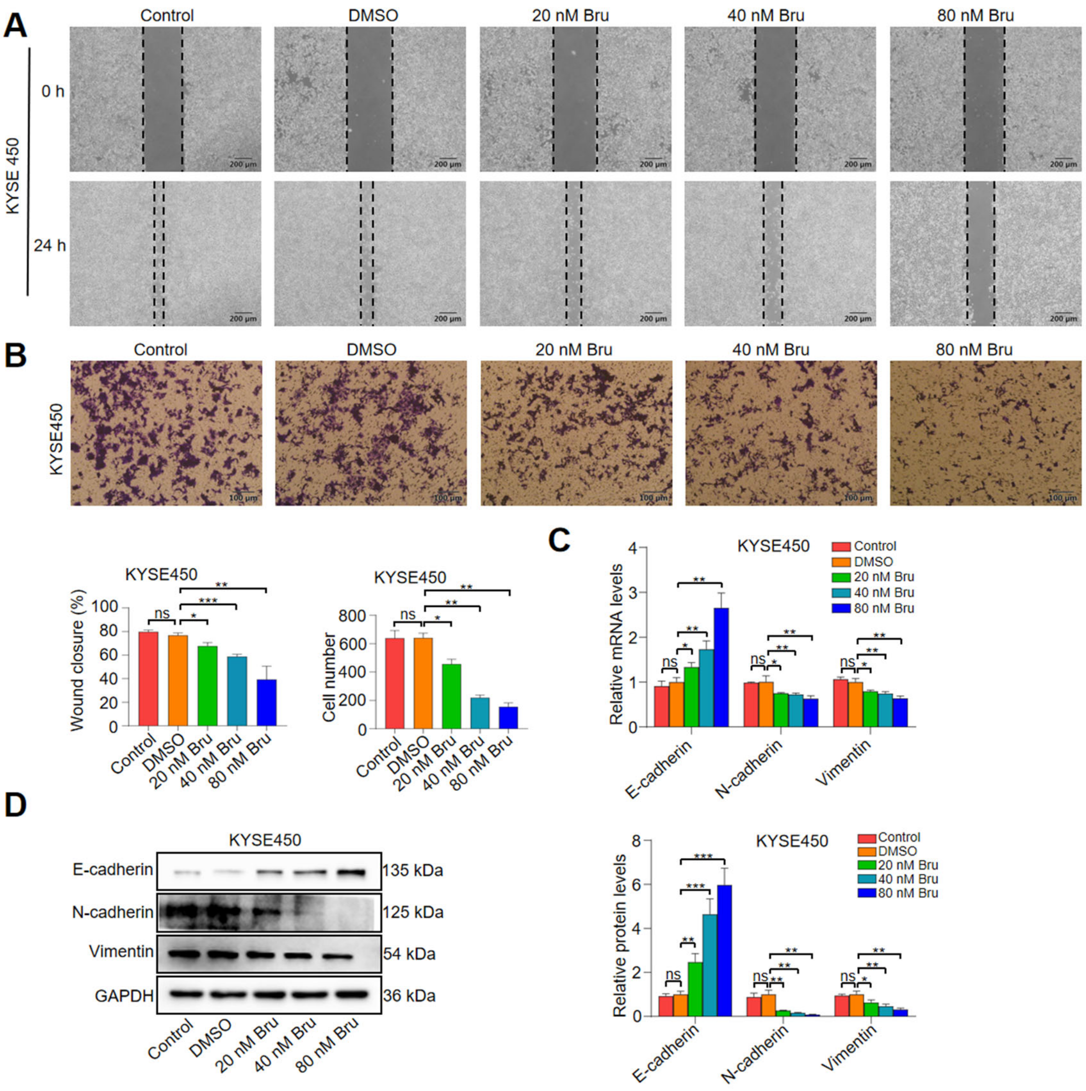

Figure S2. The effect of Bru on the migration and invasion of KYSE450 cells. (A, B) Cell migration and invasion were evaluated by wound healing and Transwell assay, quantification analysis was presented on the right. (C, D) mRNA and protein levels of E-cadherin, N-cadherin and Vimentin were measured by qRT-PCR and WB assay with quantifing using ImageJ software. The results was presented as means ± SEM (n=3). $^{*}P < 0.05$, $^{**}P < 0.01$, $^{***}P < 0.001$ *vs.* DMSO group. ns, no significance.

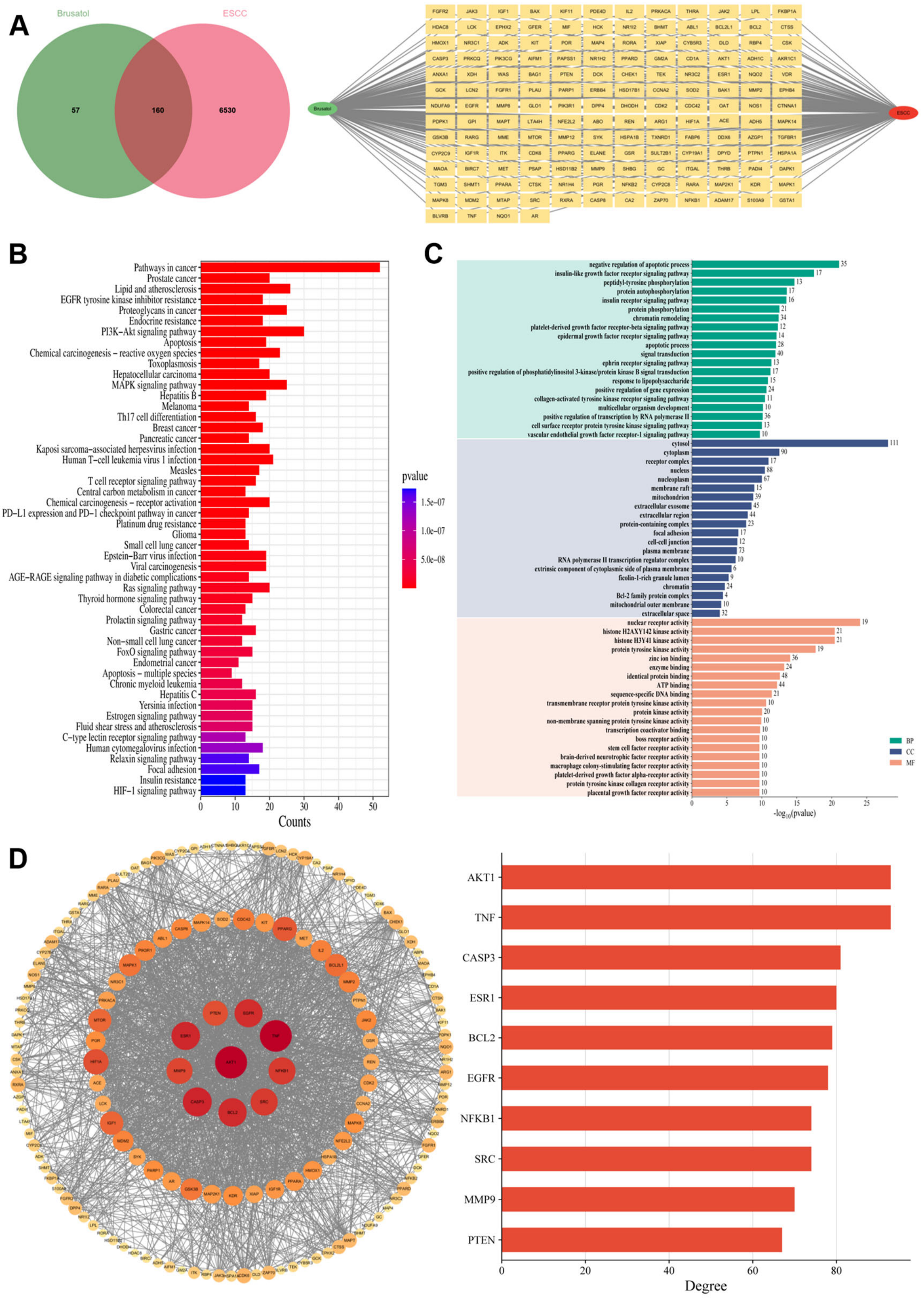

Figure S3. Network pharmacology analysis of Bru. (A) The intersection of Bru and ESCC genes sets. (B) GO enrichment for the common target genes, including biological process, cellular component, and molecular function. (C) KEGG pathway of 160 target genes. (D) PPI network of 160 duplicate targets, and top 10 targets ranked by the degree value.

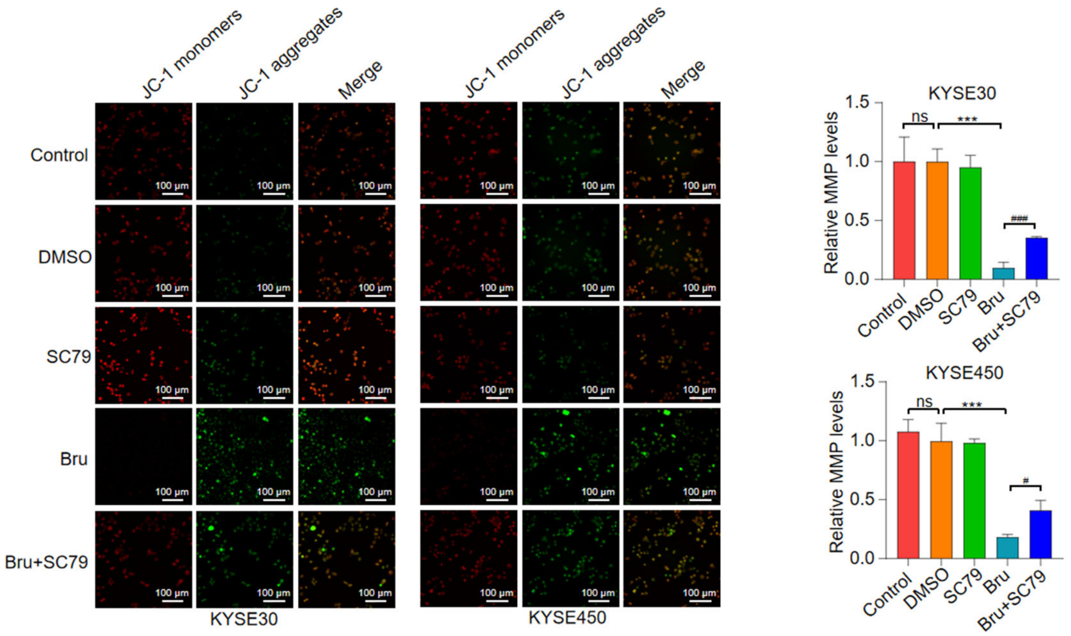

Figure S4. The effect of SC79 plus Bu on MMP of ESCC cells. MMP changes were measured via fluorescence microscopy, with quantification presented on the right. The data was presented as means ± SEM (n=3). $^{***}P < 0.001$ vs. DMSO group. $^{\#}P < 0.05$, $^{\#\#\#}P < 0.001$ vs. Bru group. ns, no significance.
