# Supplementary material for: Brusatol Inhibits Esophageal Squamous Cell Carcinoma Tumorigenesis Through Bad-Mediated Mitochondrial Apoptosis Induction and Anti-Metastasis by Targeting Akt1
Source: Biomolecules. 2025 Jun 4;15(6):812. doi: 10.3390/biom15060812 (PMC12191141; doi:10.3390/biom15060812)
Supplement: Supplementary file 1 [file biomolecules-15-00812-s001.zip › biomolecules-3563364-WB.pdf]

The following datas are the raw images of all bands in the subject, and we repeated each experiment three times.

Fig 1B

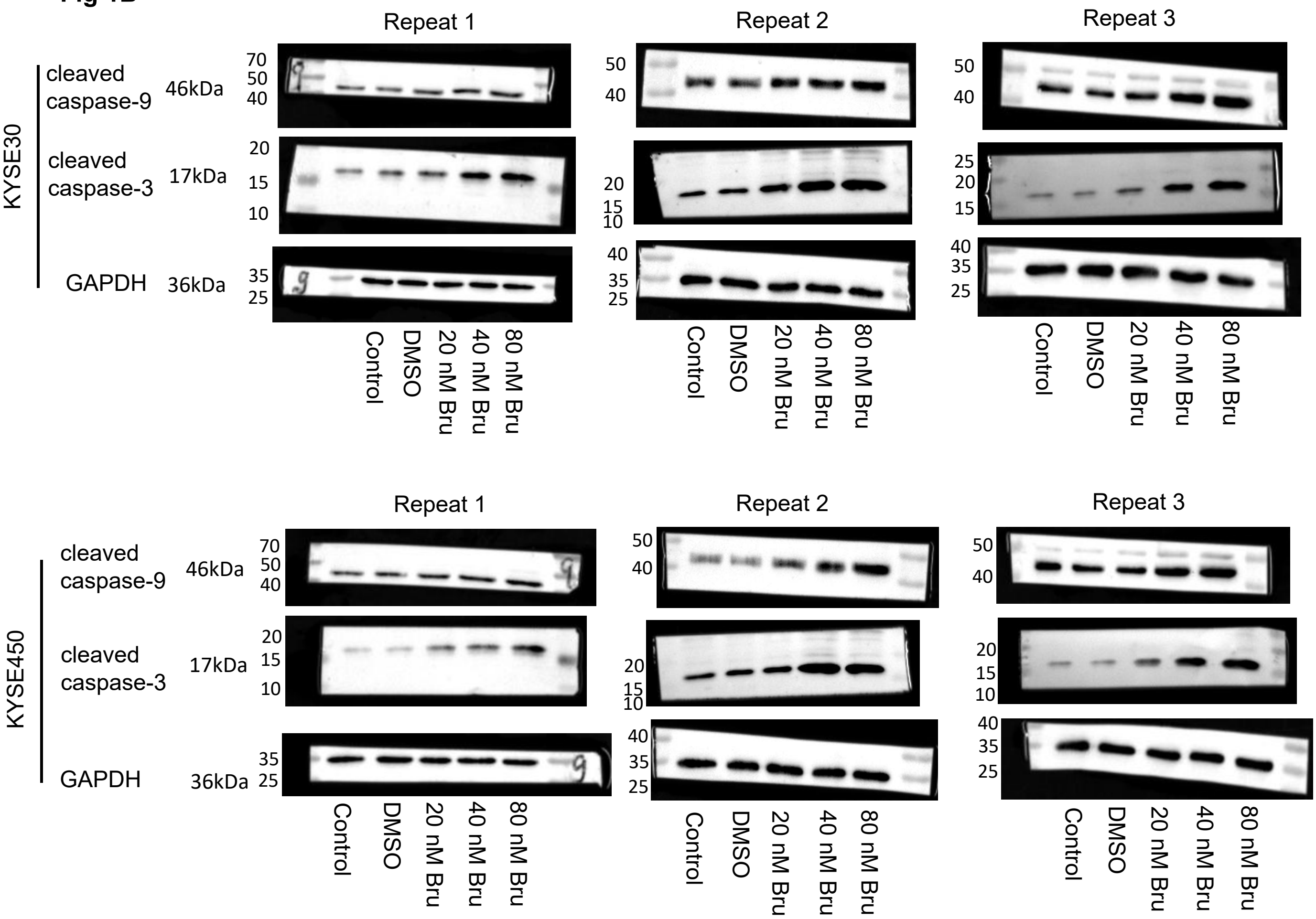

Fig 1D

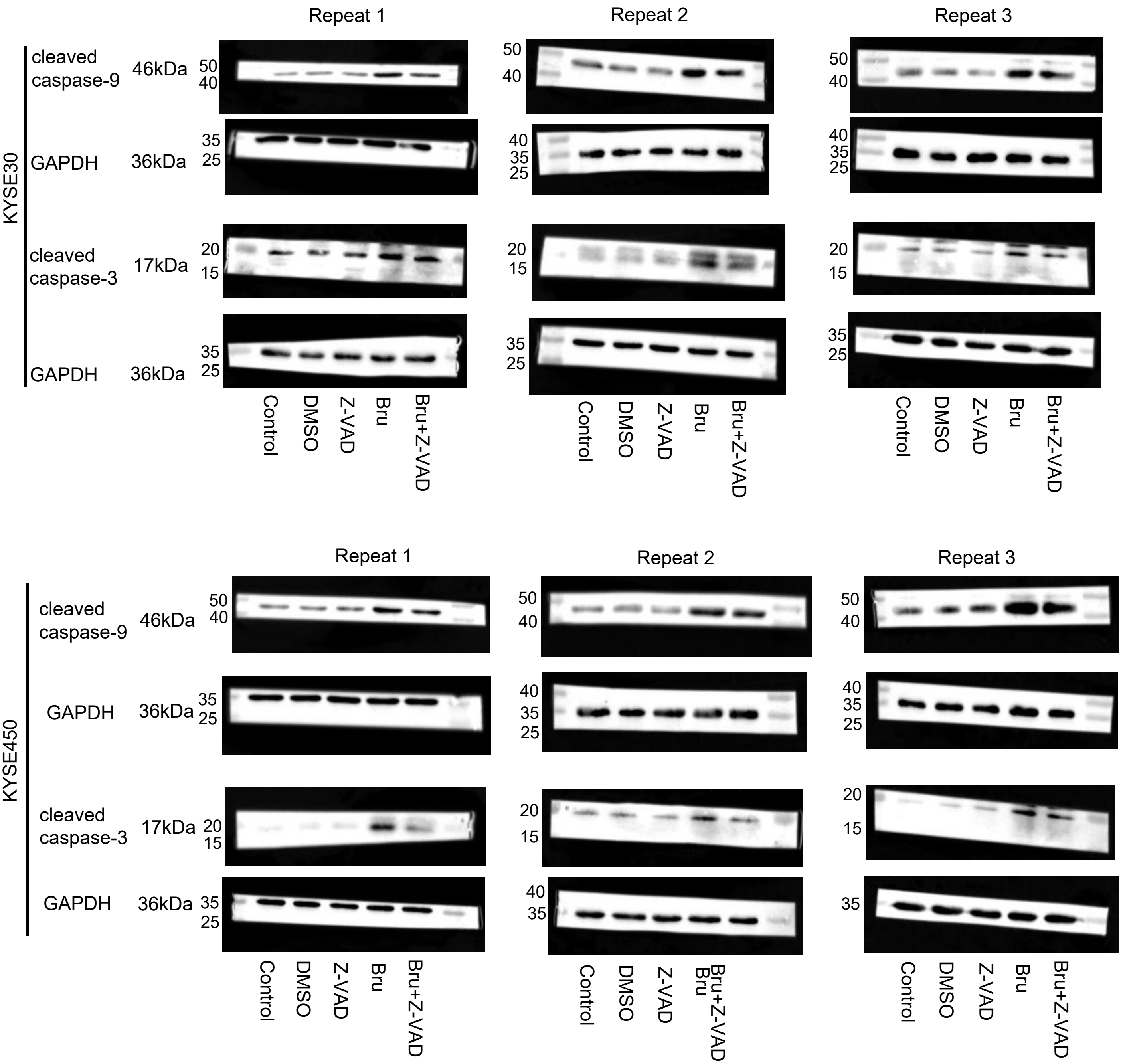
Repeat 1
Repeat 2
Repeat 3
KYSE30
cleaved caspase-9
46kDa
GAPDH
36kDa
cleaved caspase-3
17kDa
GAPDH
36kDa
50
40
35
25
20
15
Control
DMSO
Z-VAD
Bru
Bru+Z-VAD
KYSE450
cleaved caspase-9
46kDa
GAPDH
36kDa
cleaved caspase-3
17kDa
GAPDH
36kDa

Fig 2B

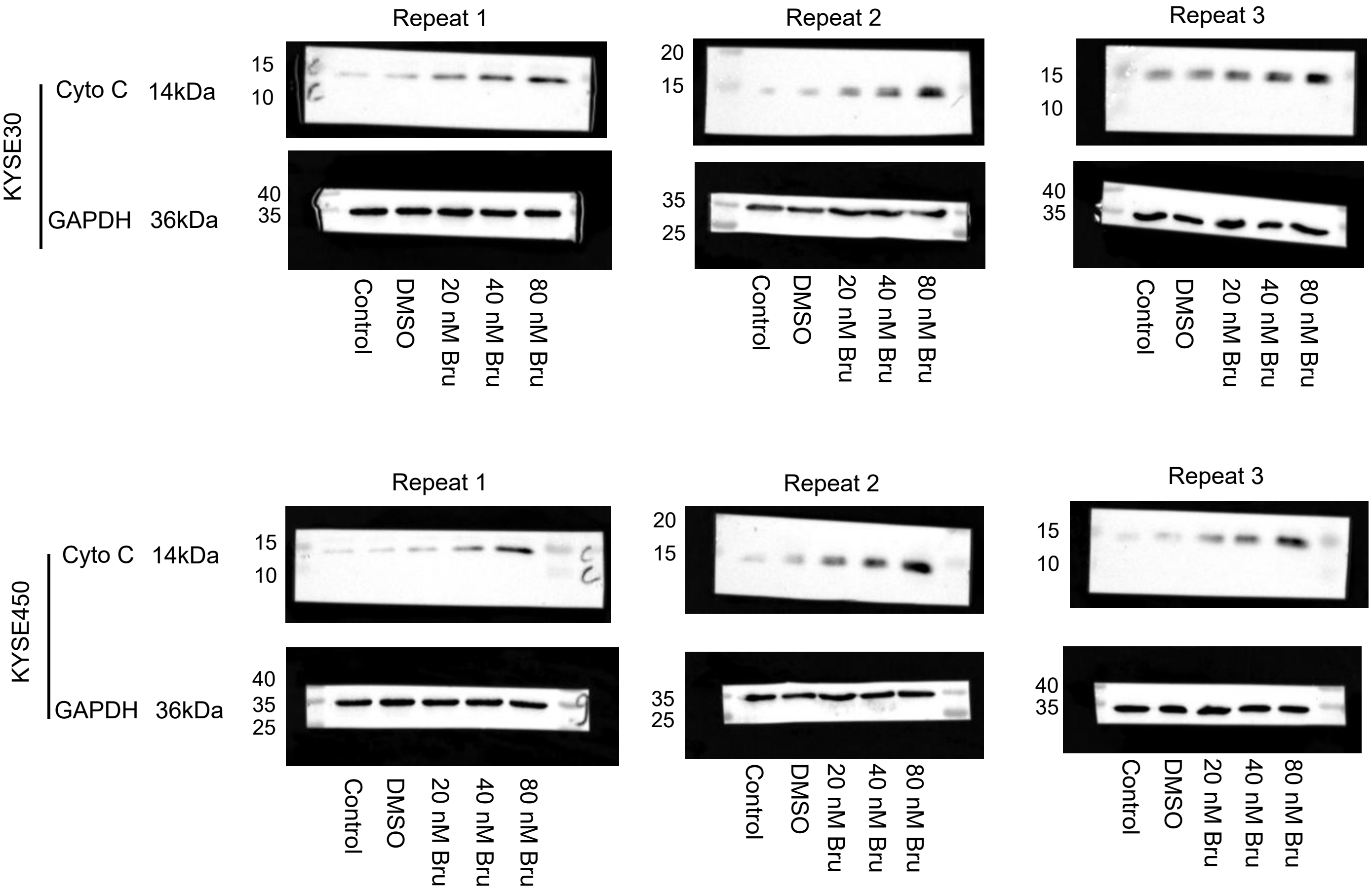
Repeat 1
Repeat 2
Repeat 3
KYSE30
Cyto C 14kDa
GAPDH 36kDa
KYSE450
Cyto C 14kDa
GAPDH 36kDa
Control
DMSO
20 nM Bru
40 nM Bru
80 nM Bru

Fig 3A

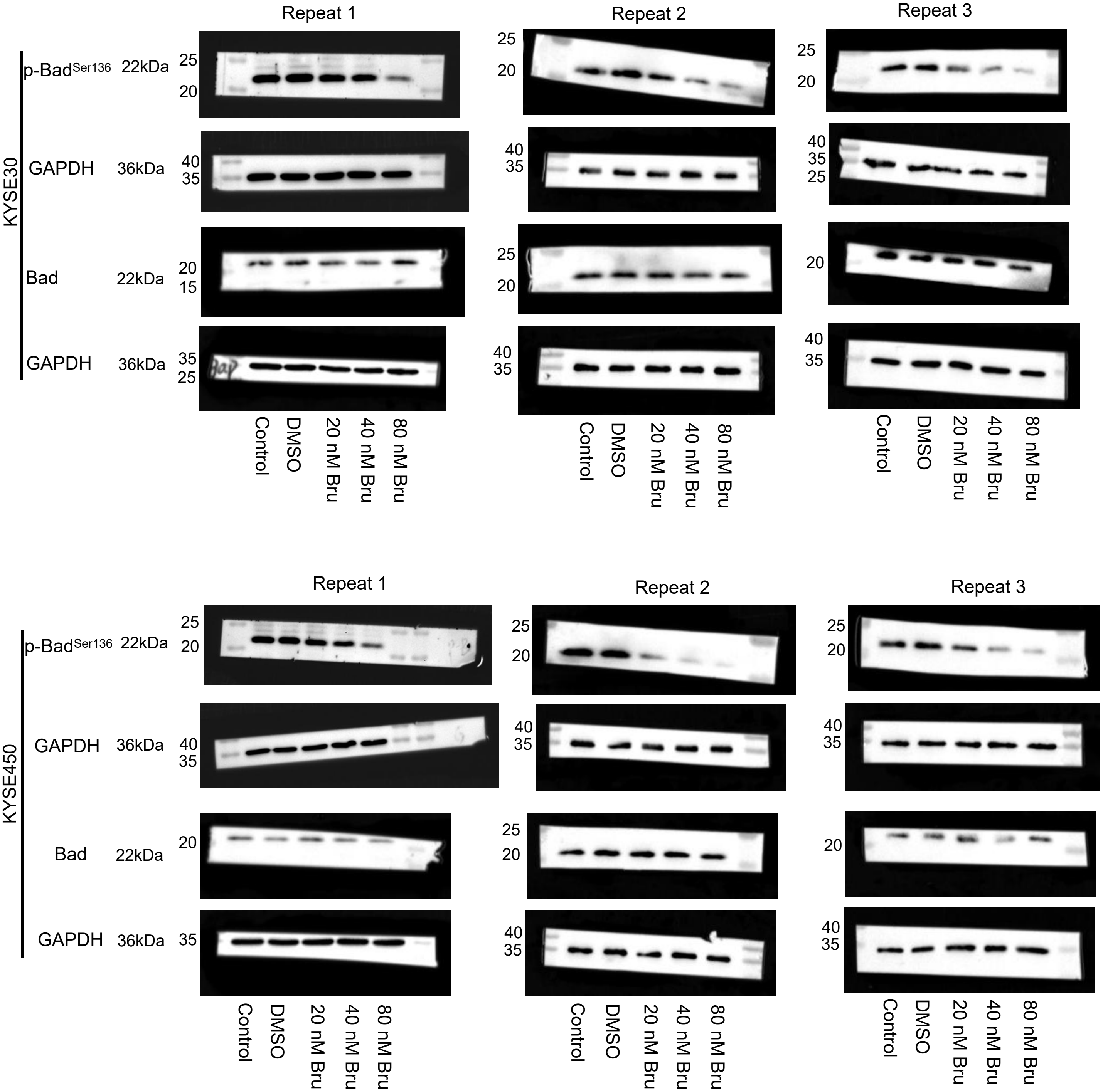

KYSE30
Repeat 1
Repeat 2
Repeat 3
p-Bad^{Ser136} 22kDa
GAPDH 36kDa
Bad 22kDa
GAPDH 36kDa
25
20
40
35
15
Control
DMSO
20 nM Bru
40 nM Bru
80 nM Bru
KYSE450
Repeat 1
Repeat 2
Repeat 3
p-Bad^{Ser136} 22kDa
GAPDH 36kDa
Bad 22kDa
GAPDH 36kDa
Control
DMSO
20 nM Bru
40 nM Bru
80 nM Bru

Fig 3C

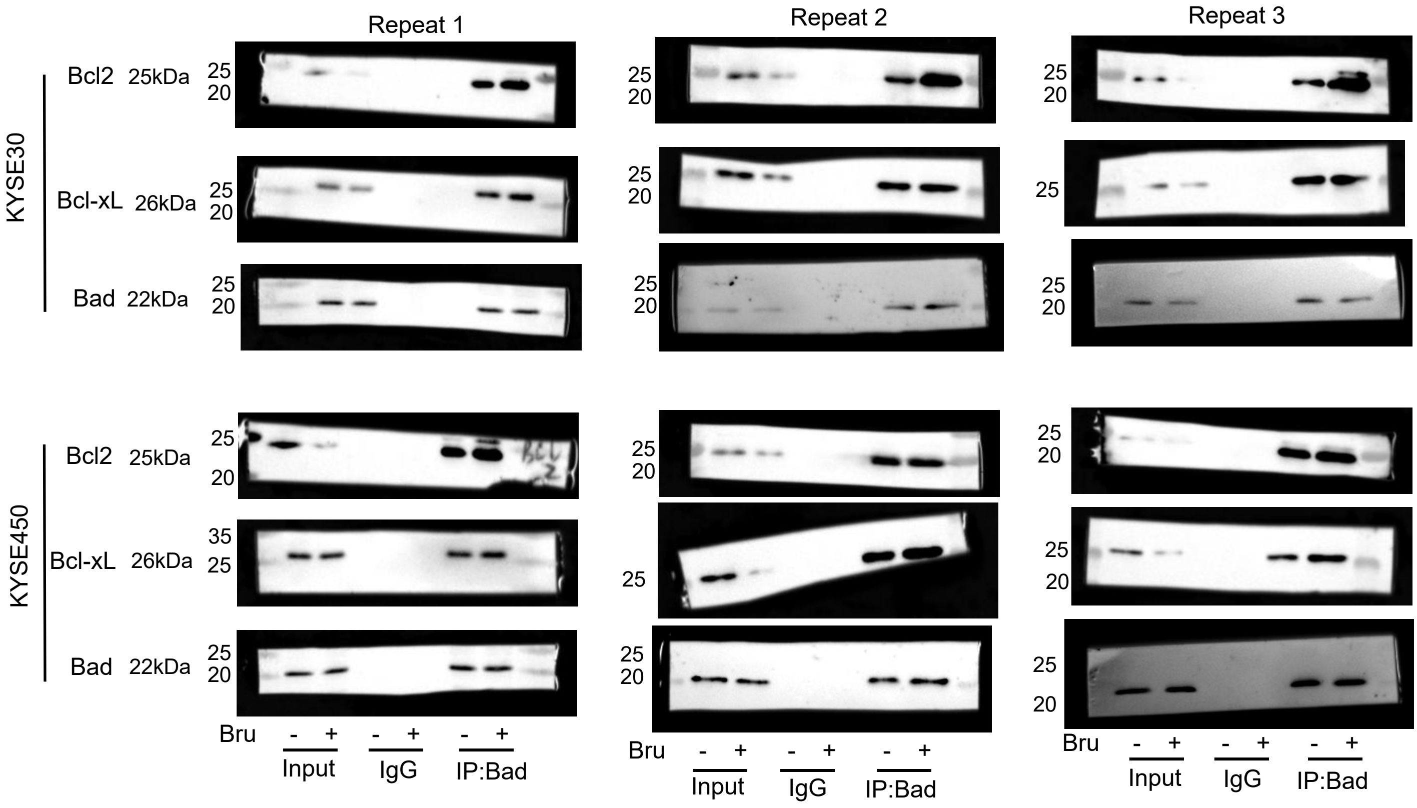
Repeat 1
Repeat 2
Repeat 3
KYSE30
Bcl2 25kDa
Bcl-xL 26kDa
Bad 22kDa
KYSE450
Bcl2 25kDa
Bcl-xL 26kDa
Bad 22kDa
25
20
35
Bru - + - + - +
Input
IgG
IP:Bad

Fig 3D

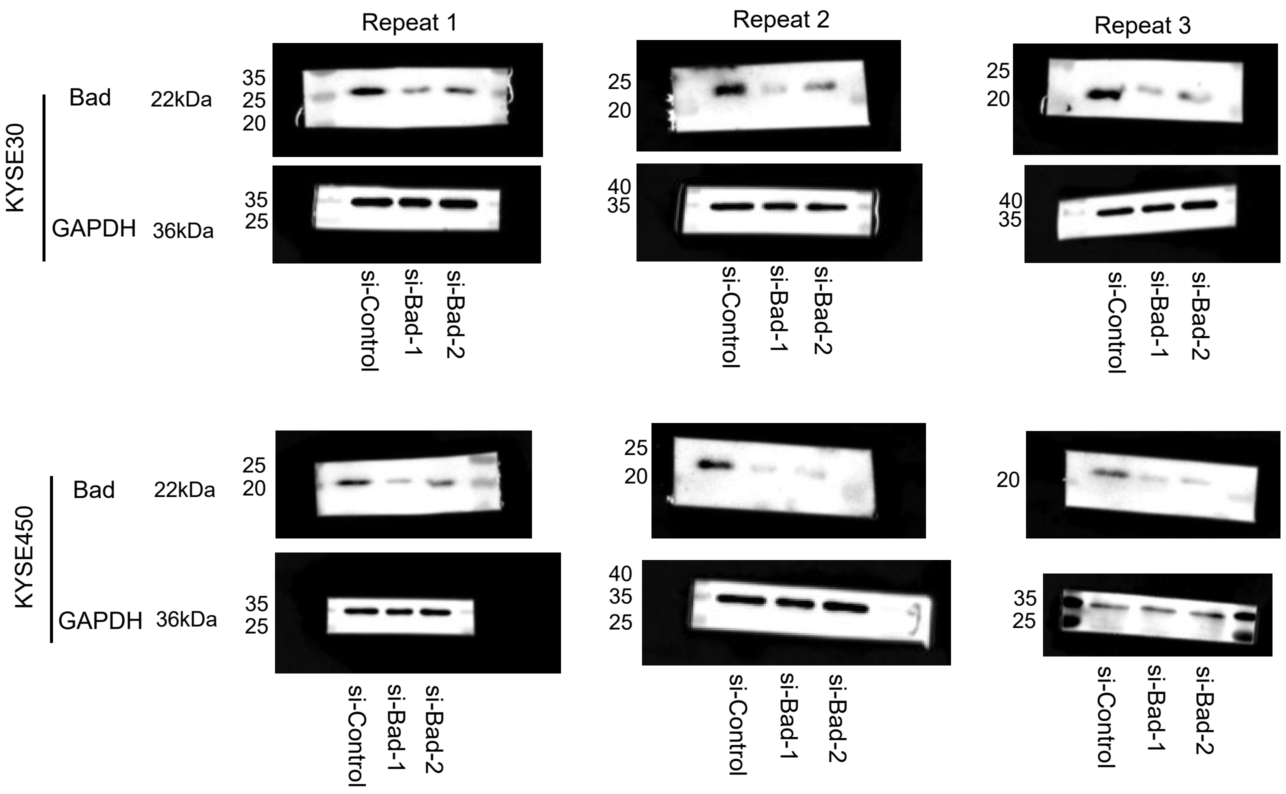
Repeat 1
Repeat 2
Repeat 3
KYSE30
Bad 22kDa
GAPDH 36kDa
KYSE450
Bad 22kDa
GAPDH 36kDa
40
35
25
20
si-Control
si-Bad-1
si-Bad-2

Fig 4D

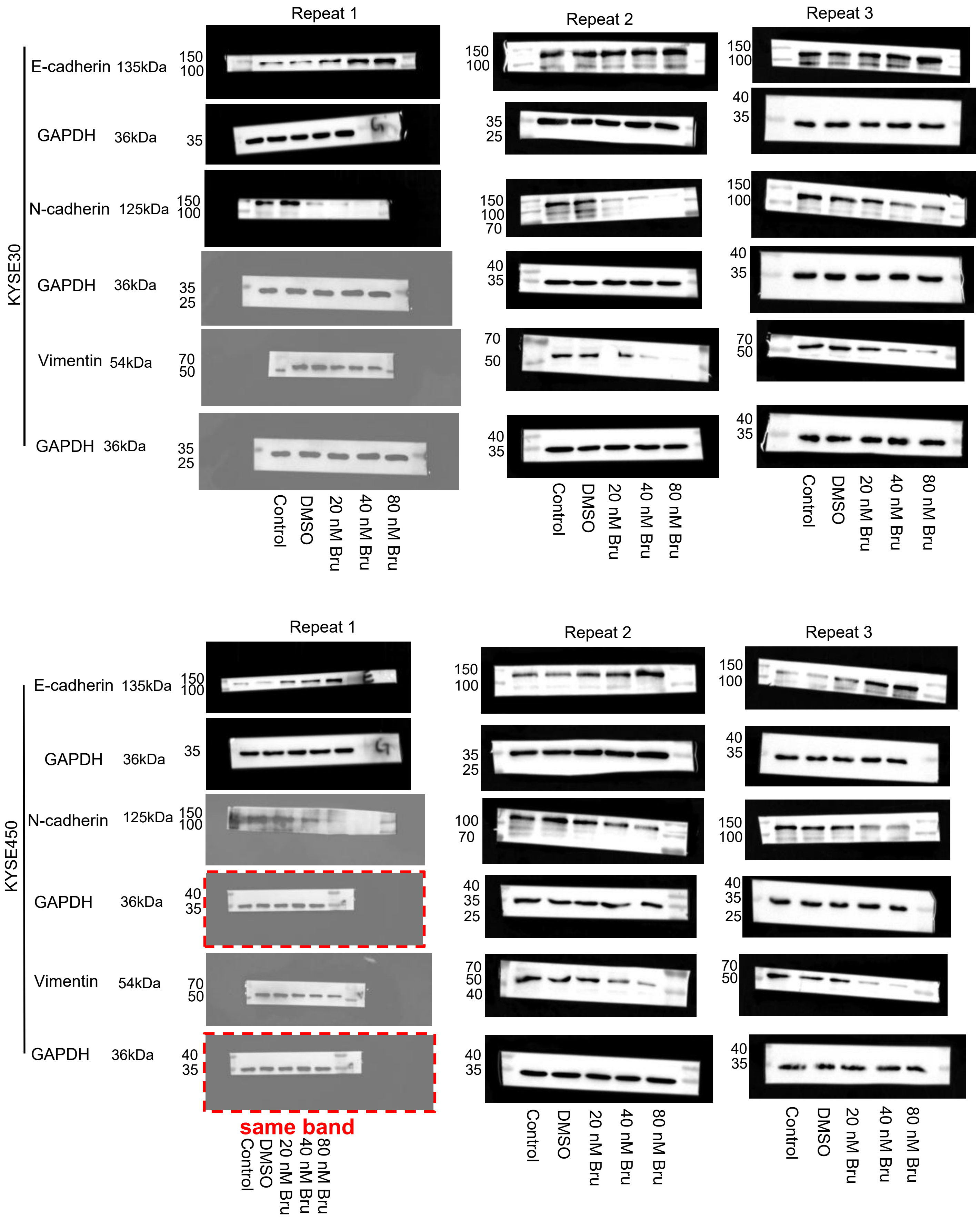

Because the same WB film cut out two different molecular weight bands, so the same GAPDH band as the internal reference.

Fig 5F

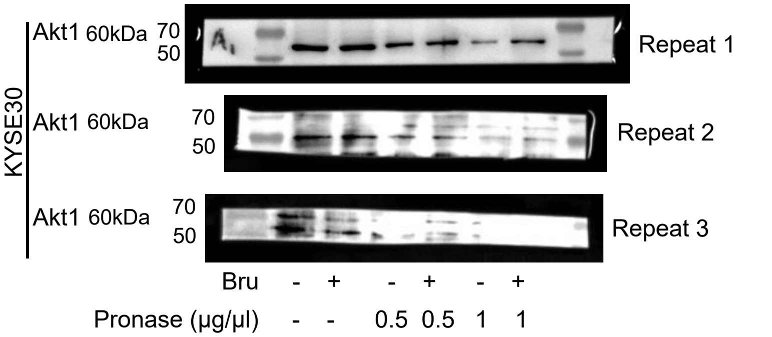
KYSE30
Akt1 60kDa
70
50
Repeat 1
Repeat 2
Repeat 3
Bru - + - + - +
Pronase (μg/μl) - - 0.5 0.5 1 1
KYSE450
Repeat 1
Repeat 2
Repeat 3
Bru - + - + - +
Pronase (μg/μl) - - 0.5 0.5 1 1

Fig 5G

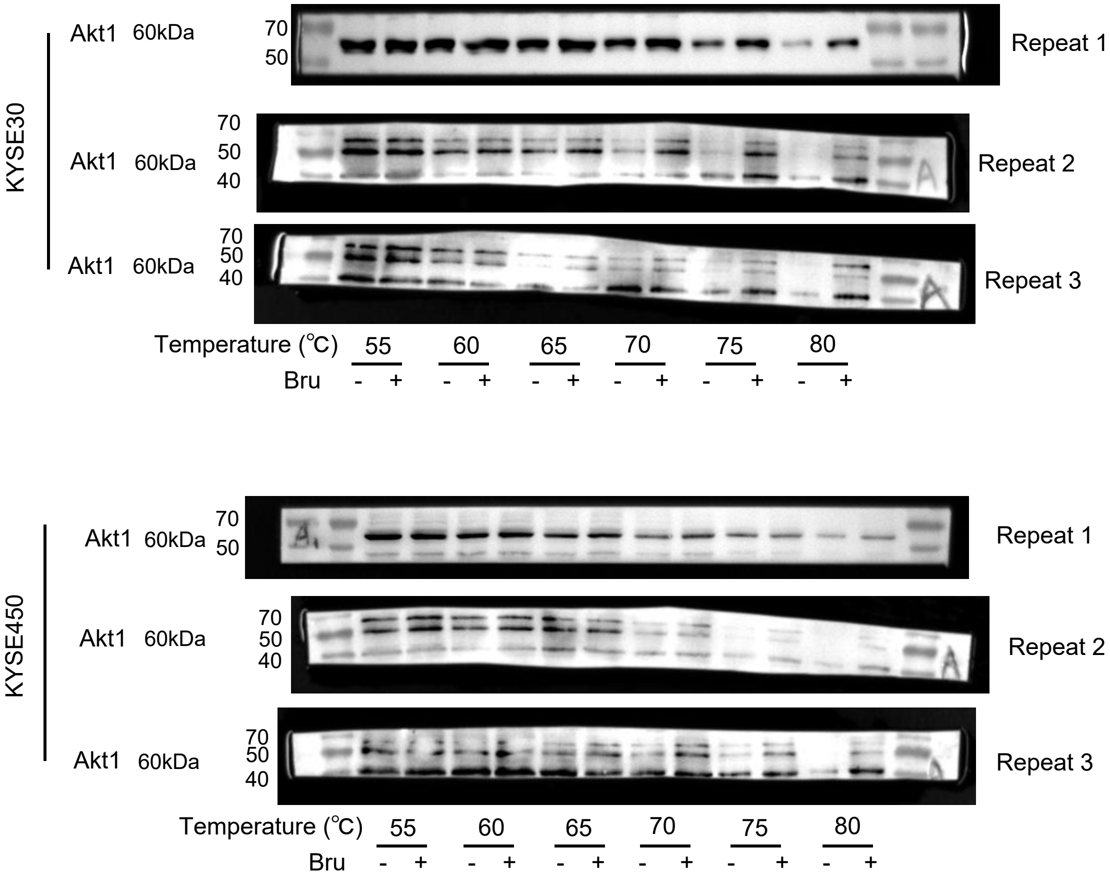
KYSE30
Akt1 60kDa
70
50
40
Repeat 1
Repeat 2
Repeat 3
Temperature (℃) 55 60 65 70 75 80
Bru - + - + - + - + - + - +
KYSE450
Repeat 1
Repeat 2
Repeat 3
Temperature (℃) 55 60 65 70 75 80
Bru - + - + - + - + - + - +

Fig 5H

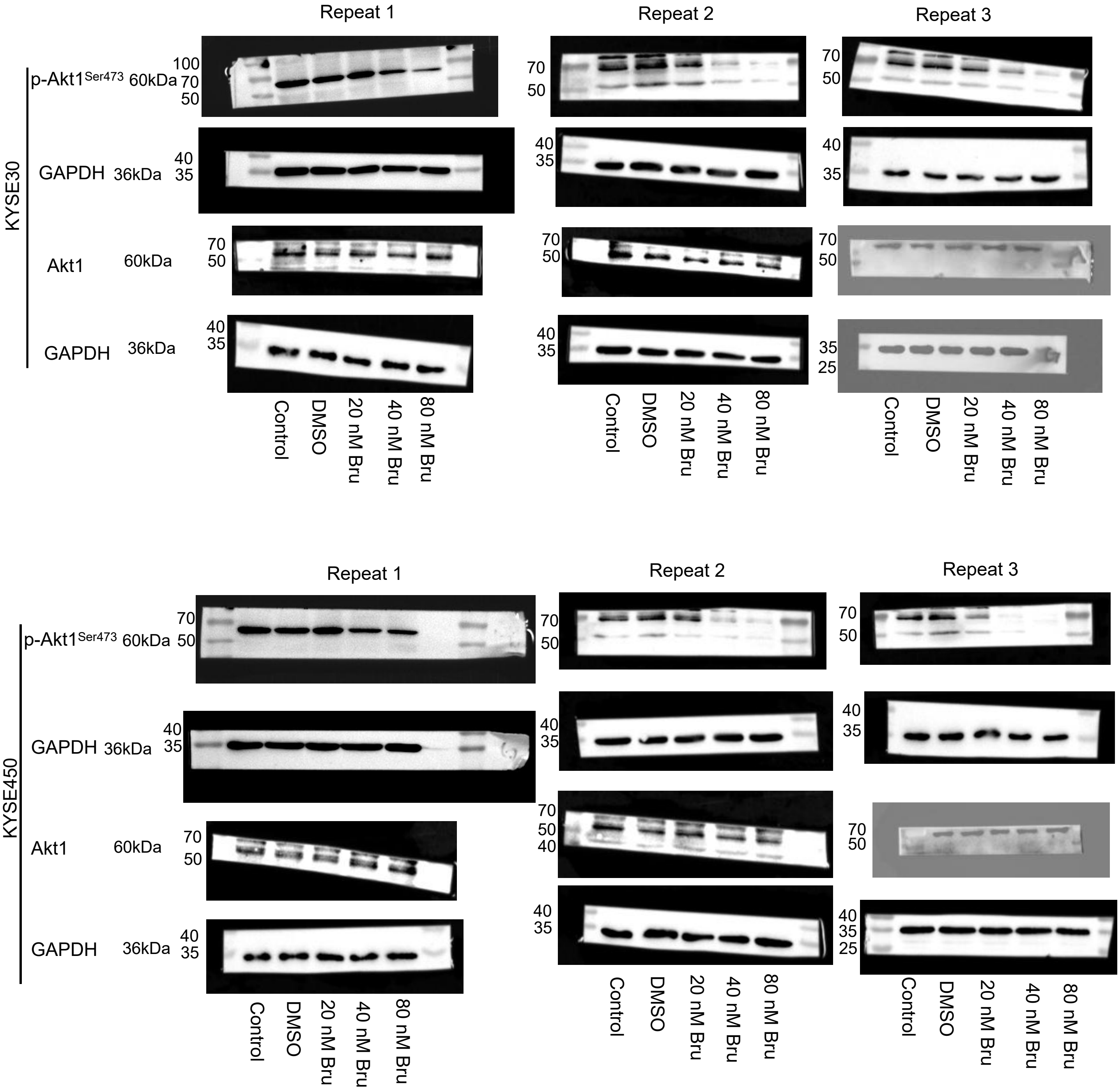
Repeat 1
Repeat 2
Repeat 3
KYSE30
p-Akt1Ser473 60kDa
GAPDH 36kDa
Akt1 60kDa
GAPDH 36kDa
100
70
50
40
35
25
Control
DMSO
20 nM Bru
40 nM Bru
80 nM Bru
KYSE450

Fig 6D

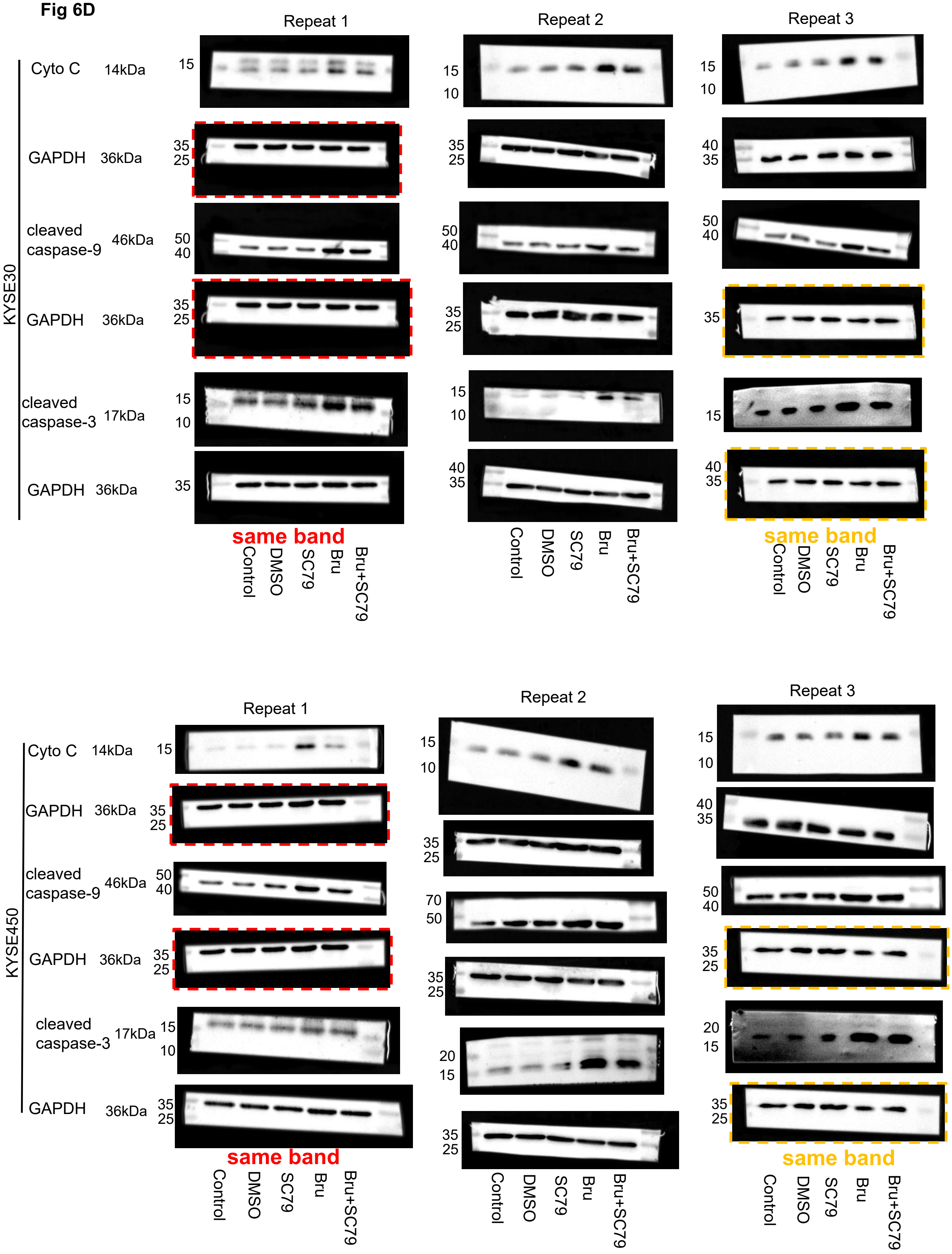

Because the same WB film cut out two different molecular weight bands, so the same GAPDH band as the internal reference.

Fig 6E

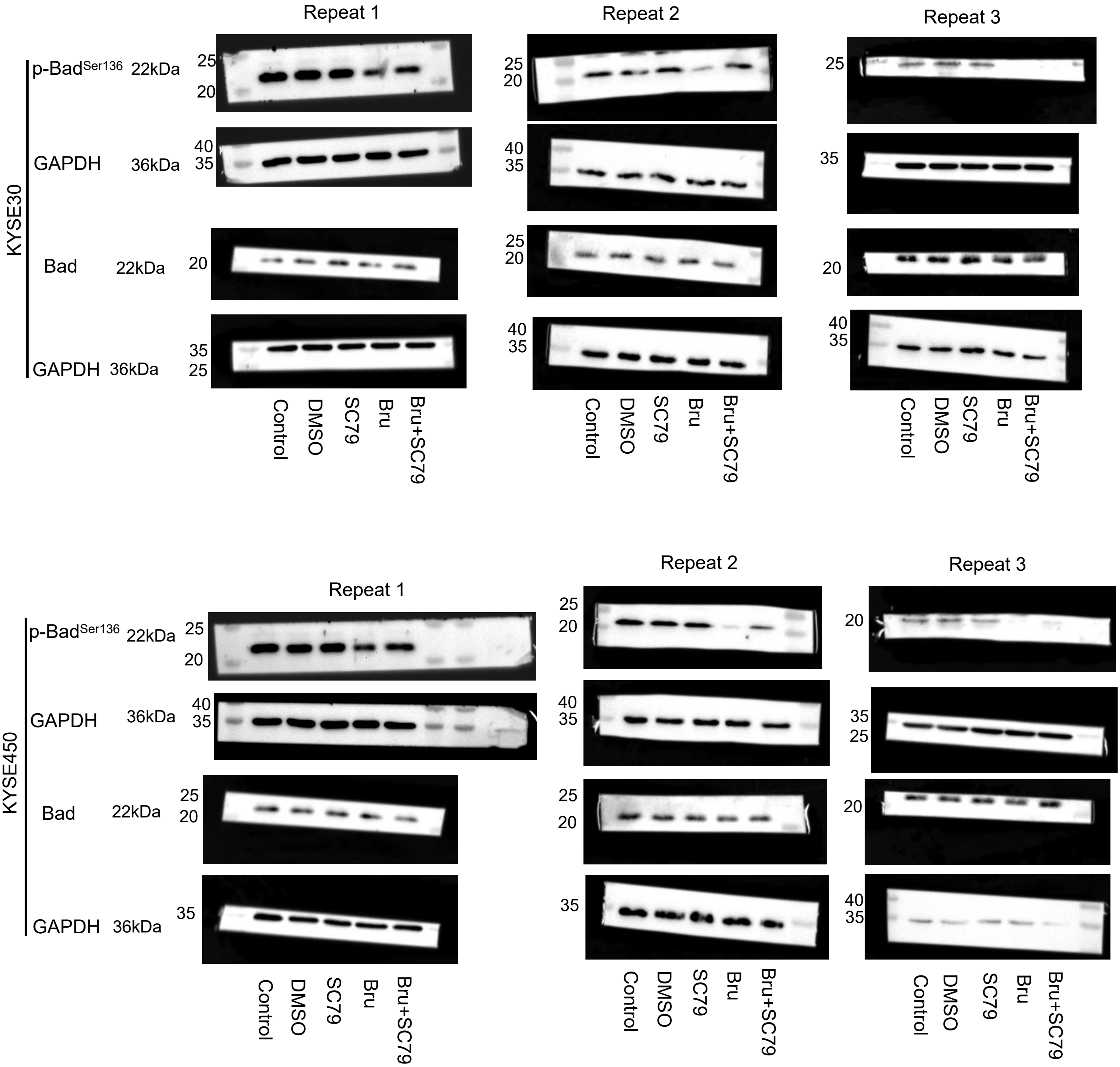
Repeat 1
Repeat 2
Repeat 3
KYSE30
p-Bad Ser136 22kDa
GAPDH 36kDa
Bad 22kDa
GAPDH 36kDa
Control
DMSO
SC79
Bru
Bru+SC79
KYSE450
p-Bad Ser136 22kDa
GAPDH 36kDa
Bad 22kDa
GAPDH 36kDa

FigS1A

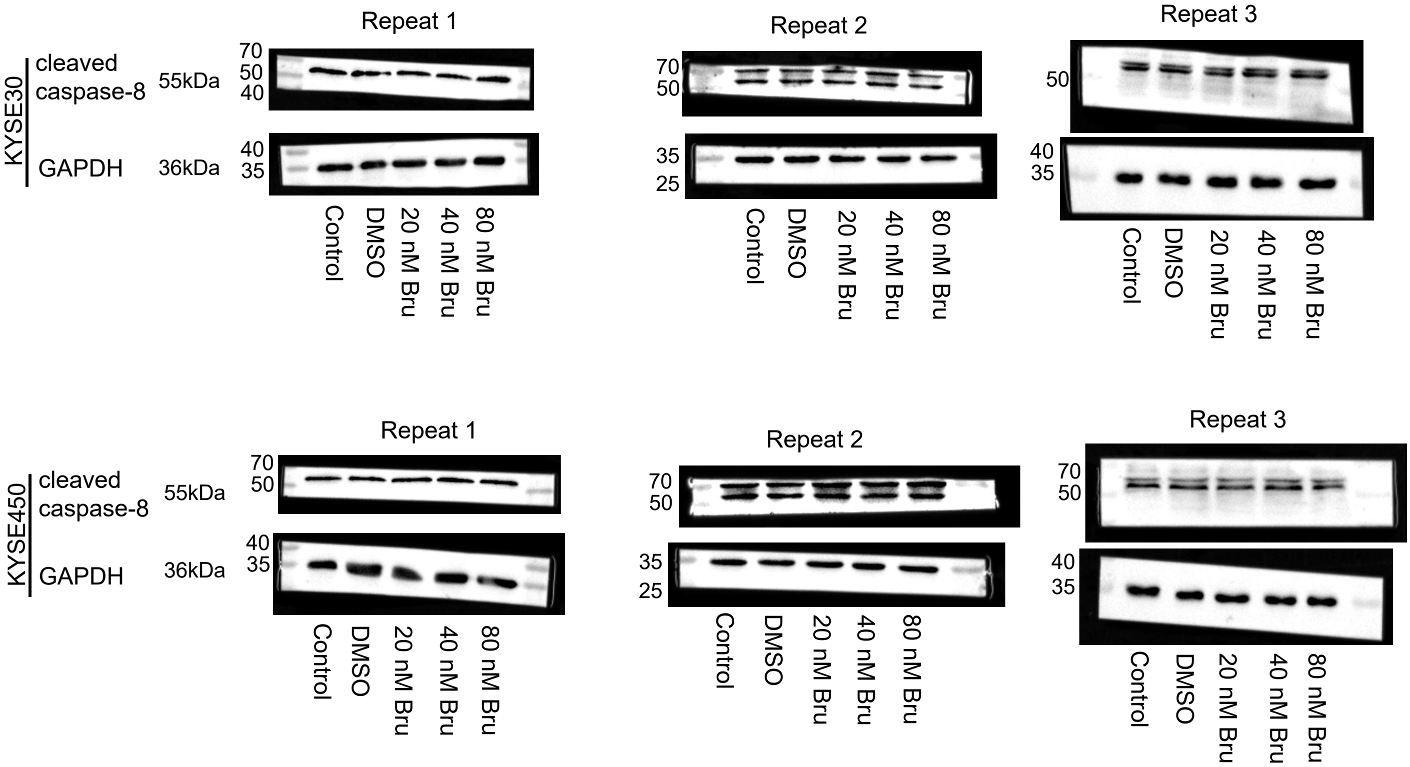
Repeat 1
Repeat 2
Repeat 3
KYSE30
cleaved caspase-8
55kDa
GAPDH
36kDa
70
50
40
35
25
Control
DMSO
20 nM Bru
40 nM Bru
80 nM Bru
KYSE450
