# Supplementary material for: Brusatol Inhibits Esophageal Squamous Cell Carcinoma Tumorigenesis Through Bad-Mediated Mitochondrial Apoptosis Induction and Anti-Metastasis by Targeting Akt1
Source: Biomolecules. 2025 Jun 4;15(6):812. doi: 10.3390/biom15060812 (PMC12191141; doi:10.3390/biom15060812)
Supplement: Supplementary file 1 [file biomolecules-15-00812-s001.zip › Table S1.pdf]

Table S1. Sequences of the primers used in the real-time PCR

Target	Forward primer sequence (5'-3')	Reverse primer sequence (5'-3')
B-actin	CACCATTGGCAATGAGCGGTTC	AGGTCTTTGCGGATGTCCACGT
E-cadherin	GCCTCCTGAAAAGAGAGTGGAAG	TGGCAGTGTCTCTCCAAATCCG
N-cadherin	CCTCCAGAGTTTACTGCCATGAC	GTAGGATCTCCGCCACTGATTC
Vimentin	AGGCAAAGCAGGAGTCCACTGA	ATCTGGCGTTCCAGGGACTCAT
