# Supplementary material for: Brusatol Inhibits Esophageal Squamous Cell Carcinoma Tumorigenesis Through Bad-Mediated Mitochondrial Apoptosis Induction and Anti-Metastasis by Targeting Akt1
Source: Biomolecules. 2025 Jun 4;15(6):812. doi: 10.3390/biom15060812 (PMC12191141; doi:10.3390/biom15060812)
Supplement: Supplementary file 1 [file biomolecules-15-00812-s001.zip › Table S2.pdf]

Table S2. Antibody list

Antibody	Dilution	Vender	Catalogue Number
Akt1	1:1000	HUABIO	Cat.No.ET1609-47
Bad	1:1000	Zenbio	Cat.No.R23582
Bcl-2	1:1000	Zenbio	Cat.No.R23309
Bcl-xL	1:1000	Zenbio	Cat.No.R23603
cleaved-caspas3	1:1000	Zenbio	Cat.No.R23727
cleaved-caspas8	1:1000	MCE	Cat.No.HY-P80624
cleaved-caspas9	1:1000	Selleckchem	Cat.No.A5074
Cytochrome C（Cyt C）	1:1000	Bimake	Cat.No.A5184
E-cadherin	1:1000	Zenbio	Cat.No.R22490
GAPDH	1:5000	Zenbio	Cat.No.301341
N-cadherin	1:1000	Zenbio	Cat.No.R380671
pAkt1^{ser473}	1:1000	Cell Signaling	Cat.No.9018T
pBad^{ser136}	1:1000	Zenbio	Cat.No.R310026
Vimentin	1:1000	Zenbio	Cat.No.R22775
Goat Anti-mouse lgG/HRP	1:5000	Zenbio	Cat.No.511203
Goat Anti-rabbit lgG/HRP	1:5000	Zenbio	Cat.No.511103
HRP-conjugated igG Fraction Monoclonal Mouse Anti-Rabbit igG, Light Chain Specific	1:500	Proteintech	Cat.No.SA00001-7L
